# TTC17 is an endoplasmic reticulum resident TPR-containing adaptor protein

**DOI:** 10.1016/j.jbc.2023.105450

**Published:** 2023-11-08

**Authors:** Nathan P. Canniff, Jill B. Graham, Kevin P. Guay, Daniel A. Lubicki, Stephen J. Eyles, Jennifer N. Rauch, Daniel N. Hebert

**Affiliations:** 1 Program in Molecular and Cellular Biology, University of Massachusetts Amherst, USA; 2 Department of Biochemistry and Molecular Biology, University of Massachusetts Amherst, USA; 3 Institute for Applied Life Sciences, Mass Spectrometry Center, University of Massachusetts Amherst, USA

**Keywords:** adaptor protein, cell compartmentalization, chaperone, endoplasmic reticulum (ER), endoplasmic reticulum stress (ER stress), ER quality control, intracellular trafficking, molecular chaperones, protein trafficking, secretion, TPR, UGGT

## Abstract

Protein folding, quality control, maturation, and trafficking are essential processes for proper cellular homeostasis. Around one-third of the human proteome is targeted to the endoplasmic reticulum (ER), the organelle that serves as entrance into the secretory pathway. Successful protein trafficking is paramount for proper cellular function and to that end there are many ER resident proteins that ensure efficient secretion. Here, biochemical and cell biological analysis was used to determine that TTC17 is a large, soluble, ER-localized protein that plays an important role in secretory trafficking. Transcriptional analysis identified the predominantly expressed protein isoform of *TTC17* in various cell lines. Further, TTC17 localizes to the ER and interacts with a wide variety of chaperones and cochaperones normally associated with ER protein folding, quality control, and maturation processes. TTC17 was found to be significantly upregulated by ER stress and through the creation and use of *TTC17*^*−/−*^ cell lines, quantitative mass spectrometry identified secretory pathway wide trafficking defects in the absence of TTC17. Notably, trafficking of insulin-like growth factor type 1 receptor, glycoprotein nonmetastatic melanoma protein B, clusterin, and UDP-glucose:glycoprotein glucosyltransferase 1 were significantly altered in H4 neuroglioma cells. This study defines a novel ER trafficking factor and provides insight into the protein–protein assisted trafficking in the early secretory pathway.

The endoplasmic reticulum (ER) is the complex multifunctional organelle that serves as the entry to the secretory pathway for roughly one-third of the human proteome (1, 2). The responsibilities of the ER include protein folding, quality control, trafficking, and calcium and lipid homeostasis (3, 4). It is composed of a single contiguous membrane and lumen that can extend from the nuclear envelope to the plasma membrane. The ER can be broadly divided into two distinct regions: the ribosomal studded and perinuclear rough ER with a sheet-like morphology and the peripheral tubular smooth ER network devoid of ribosomes (5). The ER contains additional subdomains that facilitate specialized roles such as protein translocation (translocon), vesicular transport at ER exit sites (ERES), or ER-associated degradation (ERAD) sites for the dislocation and ubiquitination to turnover terminally misfolded proteins by cytoplasmic proteasomes (6, 7).

The functional organization of multiprotein complexes facilitates the numerous roles the ER performs. Multiprotein complexes can be nucleated by scaffolding or adaptor proteins. Tetratricopeptide repeats (TPRs) are protein–protein interaction motifs commonly found in adaptors proteins that serve a variety of roles (8). The largest subgroup of the “human chaperone” is comprised of TPR-containing proteins that help to organize chaperone networks (9). TPRs consist of a degenerate 34-aa consensus sequence composing two antiparallel α-helices (8). They are frequently found in clusters of three or four consecutive repeats, which tend to recognize distinct substrate epitopes, while longer clusters of up to sixteen support more promiscuous client interactions.

A well-characterized example of TPR-mediated scaffolding is the multiprotein complex facilitated by the cytosolic Hsp70/90-organizing protein (Hop) (10). Hop contains three sets of TPR domains consisting of three TPRs each. A recent cryo-EM structure of the glucocorticoid receptor loading complex depicts two Hsp70s, a dimeric Hsp90 and the substrate with Hop (11). Hop mediates a functional and spatially organized unit for chaperone-assisted ligand activation, utilizing its TPR domains as specialized protein–protein interaction motifs.

A number of proteins that possess TPR or TPR-like domains are found in the ER where they serve a range of functions (12, 13, 14). Sec72 interacts with Hsp70 on the outer surface of the ER to help with posttranslational translocation of proteins into the ER in yeast (15). In contrast, the type I membrane protein SEL1L nucleates a retrotranslocon/ubiquitination complex to expel misfolded proteins from the ER lumen for proteasomal degradation (16, 17). The soluble ER protein ERdj6/DNAJC3/p58(IPK) serves a quality control role by interacting with BiP and misfolded proteins (18). TPRs are also proposed to be involved in substrate recognition in the ER lumen for protein modification in the case of the tetratricopeptide repeat–containing proteins for O-mannosylation or AMPylation by domain protein adenylyltransferase (13, 19, 20, 21).

We posit that additional TPR domain containing proteins may reside in the ER that help mediate the formation of multiprotein complexes. To this end, a proteome wide bioinformatic search was performed, initially predicting the localizations of the entire proteome using DeepLoc1.0 to select proteins that were potentially targeted to the ER and the secretory pathway (22) (Table S1). The putatively ER-targeted proteins were then subjected to TPR prediction using the TPRPred algorithm to identify proteins containing clusters of 3 or 4 TPRs, the common number of motifs found in adaptors and scaffolding proteins for distinct partners (10, 12, 23, 24). Through this *in silico* search, we discovered TTC17, a large soluble protein that possesses a putative N-terminal signal sequence along with two sets of TPRs, one comprised of three repeats and another of four (Fig. 1*A*).

**Figure 1:**
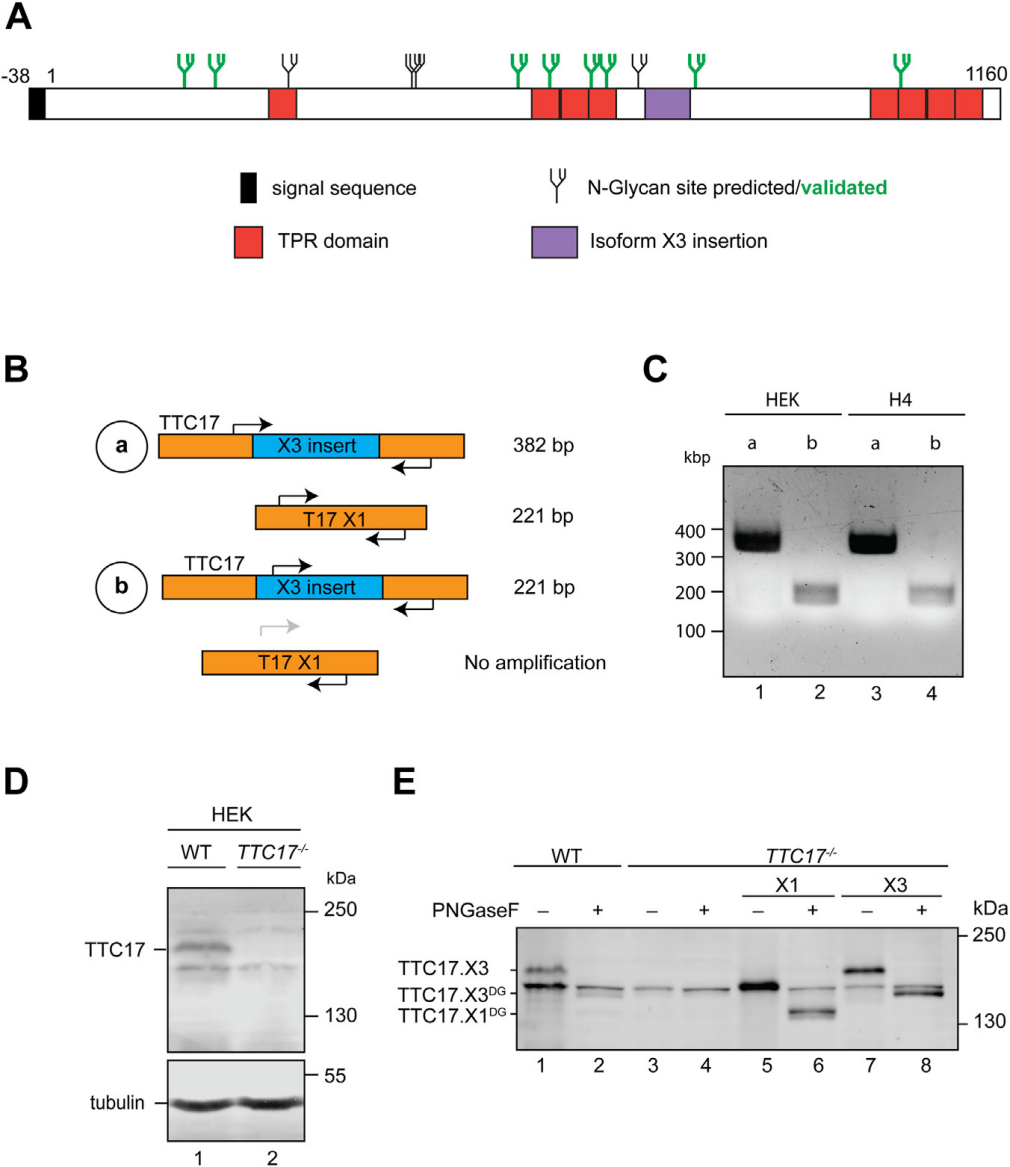
**TTC17.X3 is the predominantly expressed transcriptional and translational isoform.***A*, a putative topology map of TTC17 with signal sequence (*black*), TPR domains (*red*), N-glycan sites depicted as *small black* (predicted) *or green* (verified by MS) *branched structures*. *B*, TTC17 has two large transcripts, TTC17.X1 and TTC17.X3. The TTC17.X3 has an insertion (*blue*) but is otherwise identical to the TTC17.X1 transcript. PCRs were designed to identify which transcript is expressed. *C*, RNA was harvested from HEK cells and reverse transcribed to copy DNA, followed by PCR with indicated primers. A presentative agarose gel is displayed. *D*, HEK cells were used to generate a *TTC17*^*−/−*^ CRISPR-Cas9–edited cell line. A representative blot of the cell line is shown with the indicated band absent in the knockout. Validation *via* immunoblot using an anti-TTC17 antibody. *E*, indicated HEK cells were left untreated or transfected with either *TTC17.X1* or *TTC17.X3* copy DNA. Cells were lysed 24 h posttransfection, and the samples were then subjected to a deglycosylation assay with PNGaseF (lanes 2, 4, 6, 8). Samples were analyzed by SDS-PAGE and immunoblotted with anti-TTC17 antibody. MS, mass spectrometry; TPR, tetratricopeptide repeat.

Herein, TTC17 was rigorously characterized *in silico*, biochemically, and cellularly to elucidate its role in the secretory pathway. TTC17 is a TPR-containing protein localized to the ER—specifically, it may be localized to ERES given its interactions with the low expressed TMEM131 and transport and Golgi organization protein 1 homolog. TTC17 is upregulated both transcriptionally and translationally when subjected to a wide range of ER stress conditions, especially glycan synthesis and secretory trafficking inhibition. The absence of TTC17 leads to various trafficking defects, illustrated both in targeted assays as well as large scale secretory pathway quantitative mass spectrometry (MS). Thus, our work reveals the role of a new, soluble trafficking factor that facilitates proper protein secretion.

## Results

### TTC17-X3 is the predominant isoform expressed in HEK cells

There are multiple *TTC17* isoforms reported in the National Center for Biotechnology Information Protein Database with the UniProt canonical isoform annotated as isoform 1 (TTC17.X1). *TTC17* transcript variants present in HEK293T cells were determined by PCR on a copy DNA (cDNA) library generated from total mRNA. Sequencing the product identified the HEK293T sequence as the National Center for Biotechnology Information TTC17 isoform 3 (TTC17.X3), which corresponds to a 57 amino acid insertion in isoform 1 (Fig. 1*A*). A PCR strategy was devised to identify the predominantly expressed transcript of TTC17 in WT HEK293-EBNA1-6E (HEK) and H4 neuroglioma cells (Fig. 1*B*). PCR scheme A would generate different size products for the two isoforms: *TTC17.X1* (221-bp product) and *TTC17.X3* (382 base pair product). PCR scheme B would generate a 221-bp product if *TTC17.X3* is present, while no product would be formed if *TTC17.X1* is the sole transcript as the primers are specific for the *TTC17.X3* transcript. In Figure 1*C*, when the PCR scheme A was conducted, a ∼380 base pair product was generated and PCR scheme B generated a 221 base pair product. Both results indicate the presence of *TTC17.X3* in both the HEK and H4 cells (Fig. 1*C*).

To detect TTC17 protein, a polyclonal anti-peptide antibody against amino acids 20 to 250 of TTC17 was created, which would allow for broad isoform detection as the N terminus is conserved amongst the isoforms. The anti-TTC17 antibody recognizes endogenous TTC17 in WT HEK cells; however there are multiple bands present (Fig. 1*D*). To accurately determine which band corresponds to TTC17 as well as validate the endogenous antibody, a CRISPR-edited *TTC17* KO cell line was generated in HEK cells. The ∼170-kDa protein band that corresponds to TTC17 is absent in the HEK *TTC17*^*−/−*^ cell line, validating both the TTC17 antibody and the KO cells (Fig. 1*D*). The knockout of *TTC17* was further supported by amplicon sequencing.

To confirm TTC17 protein isoform expression, HEK WT and *TTC17*^*−/−*^ cell lysates were probed with the endogenous TTC17 antibody (Fig. 1*E*, lanes 1 and 3). The larger molecular weight band corresponding to endogenous TTC17 was absent in the *TTC17*^*−/−*^ as compared to WT cell lysate. HEK *TTC17*^*−/−*^ cells were transfected with cDNA corresponding to *TTC17.X1* or *TTC17.X3* isoforms and probed with the anti-TTC17 antibody. The TTC17.X3 band migrates equivalent to that of the endogenous TTC17, whereas the TTC17.X1 isoform migrates similarly to a lower band (Fig. 1*E*, lanes 1, 5, and 7). To identify if the TTC17.X1 isoform is present and masked by the background band, lysates were treated with an endoglycosidase. Secretory proteins are commonly modified upon entrance into the ER with N-linked glycans. N-glycans are appended to Asn residues at specific acceptor site (Asn-Xxx-Ser/Thr/Cys, where Xxx ≠ Pro) by oligosaccharyltransferases (OSTs) (25). TTC17 possesses multiple N-linked glycan acceptor sites (Fig. 1*A*). If TTC17.X1 is indeed present, there should be a mobility shift down when the glycans are removed by an endoglycosidase. However, when the lysates are treated with the endoglycosidase PNGaseF, the lower molecular weight band in the lysates does not move in contrast to the large mobility shift seen in the transfected TTC17.X1 treated with PNGaseF (Fig. 1*E*, lane 6). The lower molecular weight band, therefore, corresponds to both the TTC17.X1 isoform and a background band. The *TTC17.X3* is the predominantly expressed transcript in HEK cells and thus it will be referred to herein as *TTC17*.

### TTC17 is a resident ER protein

There are conflicting reports as to the cellular localization of TTC17. An initial study suggested that TTC17 is localized in the cytoplasm where it helps organize actin for ciliogenesis (9). A more recent study used a CRISPRi screen to identify genes involved in protein trafficking placed TTC17 from HeLa cells in the Golgi where it was proposed to play a role in Golgi organization (26).

*In silico* analysis using SignalP6.0 and DeepLoc1.0 indicates that TTC17 potentially contains an unconventional N-terminal signal sequence predicted to direct it to the ER (Fig. 1*A*) (22, 23, 27, 28, 29). Signal sequences are commonly 15 to 20 amino acids in length (30). The predicted TTC17 N-terminal signal sequence is 38 amino acids (Fig. S1*A*). Earlier versions of SignalP did not identify the N terminus of TTC17 as a signal sequence given its extended length. Based on the predictive analysis with the algorithm SignalP6.0, the hydrophobic core of the signal sequence occurs from amino acid 20 to 31. This is followed by a predicted Ala-Xxx-Ala cleavage site after Ala38. Given the noncanonical nature of the predicted signal sequence of TTC17 and the discrepancy with the published results, a variety of targeting and localizations assays were performed to determine if TTC17 was targeted to the ER.

**Figure 2:**
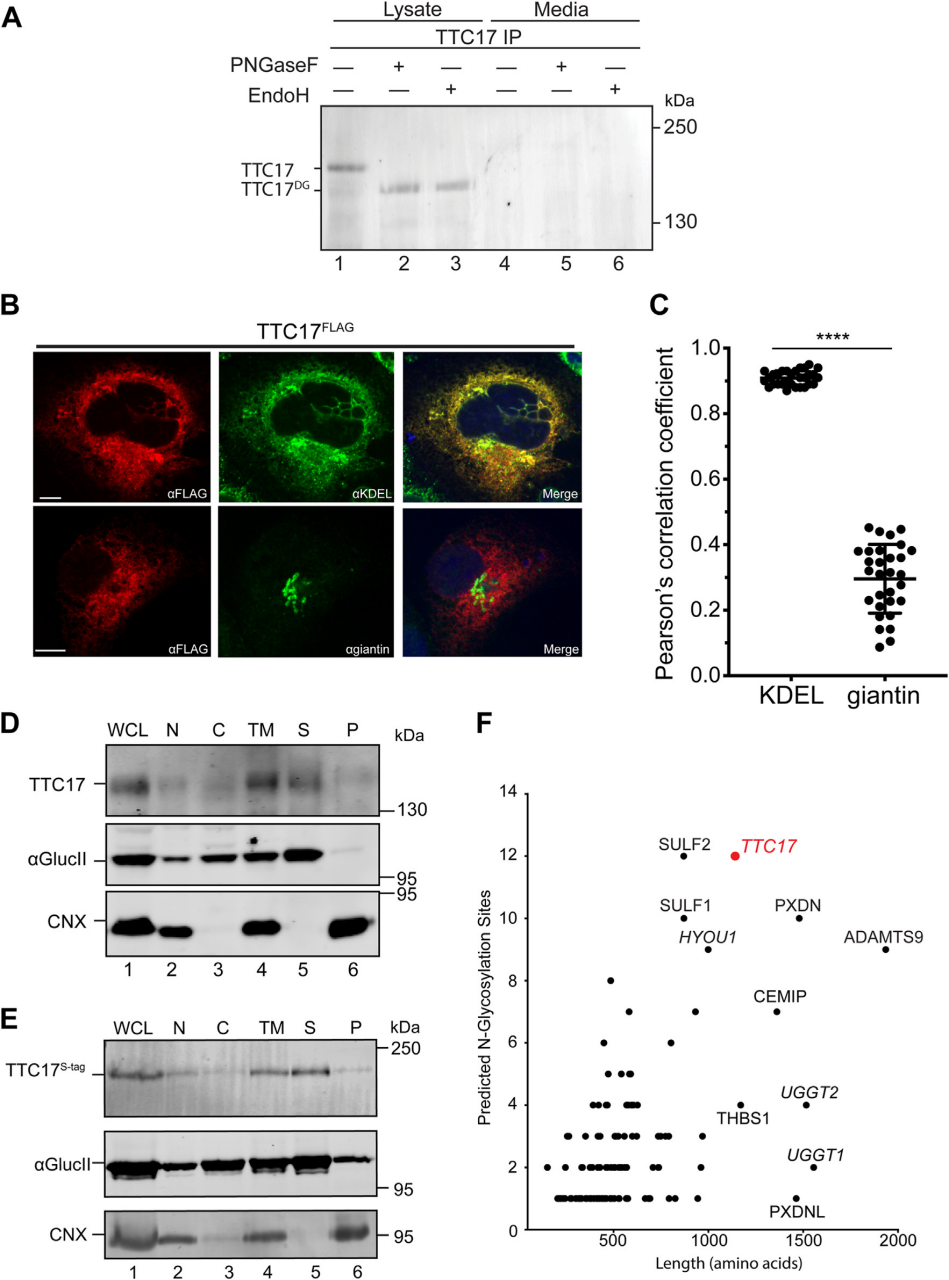
**TTC17 is a soluble ER luminal protein.***A*, HEK cells were lysed and TTC17 was immunopurified from cell lysate and media using an anti-TTC17 polyclonal antibody. Samples were then subjected to a deglycosylation assay with either PNGaseF (lanes 2 and 5) or EndoH (lanes 3 and 6) digestion as indicated. The samples were analyzed by reducing 7.5% SDS-PAGE and immunoblotted with an anti-TTC17 antibody. *B*, cellular localization of TTC17^FLAG^ was investigated by confocal microscopy. COS7 cells were transfected with *TTC17*^*FLAG*^ copy DNA, fixed, and stained with α-FLAG (TTC17, *red*), α-KDEL (ER, *gree*n) or α-giantin (Golgi, *green*). Nuclei were visualized by 4′,6′-diamidino-2-phenylindole staining (*blue*). The scale bar represents 10 μm. *C*, colocalization of with TTC17 with the ER or Golgi markers from (*B*). Analysis of the overlap between TTC17 and organelle markers was determined by Pearson’s correlation coefficient. Pearson’s correlation coefficient were calculated for TTC17 *versus* KDEL (0.92 ± 0.02) and giantin (0.30 ± 0.11). Each group represents 30 individual cells collected over three independent biological replicates. Error bars represent the SD within the sample and ∗∗∗∗ indicates *p*-value <0.0001. *D*, CHO cells were homogenized and fractionated prior to alkaline extraction. The fractions collected were the whole-cell lysate (WCL), nucleus (N), cytosol (C), total membrane (TM), as well as the supernatant (S) and pellet (P) fractions after alkaline extraction of the TM fraction. Samples were resolved *via* reducing SDS-PAGE and analyzed by immunoblotting with antisera against TTC17, the type I membrane protein calnexin (CNX), and the soluble protein α-glucosidase II (αGlucII). *E*, HEK293T cells were transfected with *TTC17*^*S-tag*^ copy DNA and treated as in (*D*). *F*, soluble proteins with ER localization as per UniProt accessions plotted by predicted N-glycan sites and length (amino acids), with TTC17 highlighted in *red* and known ER resident proteins are *italicized*. ER, endoplasmic reticulum; KDEL, KDEL Lys-Asp-Glu-Leu.

First, endogenous TTC17 expression in HEK293T cells was monitored by immunoprecipitation (IP) followed by immunoblotting (Fig. 2*A*). TTC17 levels in cell lysates and the cell media were probed with an anti-TTC17 polyclonal antibody. A prominent TTC17 band was observed in cell lysates that migrated with a molecular weight of ∼170-kDa (Fig. 2*A*, lane 1). The background band observed in Figure 1, *D* and *E* was not seen when the sample was IP prior to immunoblotting. Furthermore, the absence of the TTC17 in the cell media suggested that TTC17 was not a secreted protein but rather a cellular retained protein.

We previously saw that TTC17 was modified by N-glycans using a PNGaseF sensitivity assay (Fig. 1*D*). PNGaseF treatment caused a significant mobility shift for TTC17 demonstrating efficient carbohydrate modification that required ER targeting (Fig. 2*A*, lanes 1 and 2). N-glycans appended to a protein upon entry into the ER by the OST are high mannose glycoforms (31). An endoglycosidase H (EndoH) sensitive form is maintained in the ER. However, upon entry to the Golgi, glycoproteins are commonly further trimmed and then modified with complex glycans that render the glycoprotein EndoH-resistant (32). TTC17 was EndoH-sensitive supporting it containing high mannose glycoforms (Fig. 2*A*, lane 3). The Endo H sensitivity of TTC17 and its absence in the media are consistent with TTC17 remaining in the cell and residing in the ER.

TTC17 cellular localization was further investigated using confocal immunofluorescence microscopy. COS7 cells are commonly used for imaging due to their thinness (33). COS7 cells were transfected with TTC17^FLAG^ cDNA and staining was compared against ER (KDEL) and Golgi (giantin) markers. TTC17^FLAG^ colocalized with the ER marker as shown by a Pearson’s correlation coefficient approaching 1, while a poor overlap was observed with the Golgi marker (Fig. 2, *B* and *C*). Taken together, TTC17 is an ER resident protein as demonstrated by glycosylation, glycosidase sensitivity, and confocal immunofluorescence microscopy.

### TTC17 is a highly N-glycosylated soluble ER protein

N-terminal sequences can act as signal anchor sequences to create type II or polytopic membrane proteins. Alternatively, if the signal sequence is cleaved and the remainder of protein lacks transmembrane domains, it would produce a soluble protein. Hydrophobicity analysis of TTC17 with DeepTMHMM found that the only hydrophobic sequence was the N-terminal sequence that serves as the signal sequence (Fig. S1*B*) (34).Therefore, whether TTC17 was an ER membrane integrated or soluble protein was explored using alkaline membrane extractions.

TTC17 tagged with a C-terminal S-tag (TTC17^S-tag^) was transfected in HEK293T cells. These cells were homogenized in isotonic buffer and separated by centrifugation to separate nuclear (N), cytosolic (C), and total membrane (TM) fractions. The TM fraction was then subjected to treatment with an alkaline buffer to collapse the membranous structures and expel the soluble proteins upon centrifugation (35). This treatment resulted in the separation of the soluble (S) and membrane (P, pellet) fractions. TTC17 appeared in the soluble fractions when either overexpressed in HEK239T or endogenously expressed in CHO cells (Fig. 2, *D* and *E*). Therefore, the alkaline extraction assay found TTC17 to be a soluble protein localized within the endomembrane system of the cell.

TTC17 has 12 predicted N-linked glycosylation sites (Fig. 1*A*). The molecular weight of an N-linked glycan, while in the ER is 1.80 to 2.06 kDa (for Man_7_GlcNAc_2_ to Man_9_GlcNAc_2_, respectively). The removal of N-linked glycans with the glycosidase PNGaseF treatments results in a ∼26-kDa mobility shift endogenous TTC17 (Fig. 2*A*). This shift is consistent with most of the 12 predicted sites being modified.

The modified N-glycosylated sites on TTC17 were mapped by overexpression of TTC17^FLAG^ followed by IP LC-MS/MS. Cleavage by PNGaseF causes a deamidation reaction where the Asn is converted to Asp. This conversion can be visualized by LC-MS/MS analysis due to an increase in mass of ∼1 Da. Using this method, eight N-glycans were confirmed for TTC17 (Fig. 1*A*, green N-glycans and Table S2). The four missing sites are located on peptides that were not visualized, therefore at this time their modification cannot be ruled out. If all twelve glycosylation sites are modified on TTC17 as suggested by the PNGaseF treatment mobility shift (Fig. 2*A*), TTC17 would be the most highly N-glycosylated soluble resident ER protein. This was shown by our *in silico* analysis of the soluble glycoproteome where all glycosylation consensus sites on these soluble proteins that are indicated to have ER localization, as per UniProt accessions, are considered to be modified (Fig. 2*F*, ER resident proteins are italicized).

### TTC17 interactions identified by proximity labeling and co-IP

The ER is often the largest organelle in the cell and has suborganellar compartments within its overall structure (6, 25, 36, 37, 38). To confirm ER localization, possibly probe sub-organellar localization and identify putative TTC17 interactors, a proximity labeling technique was employed. This involved the use of a fusion protein between TTC17 and the promiscuous biotin ligase TurboID placed at the C terminus of TTC17 isoforms X1 and X3, followed by a 3× FLAG tag (39).

**Figure 3:**
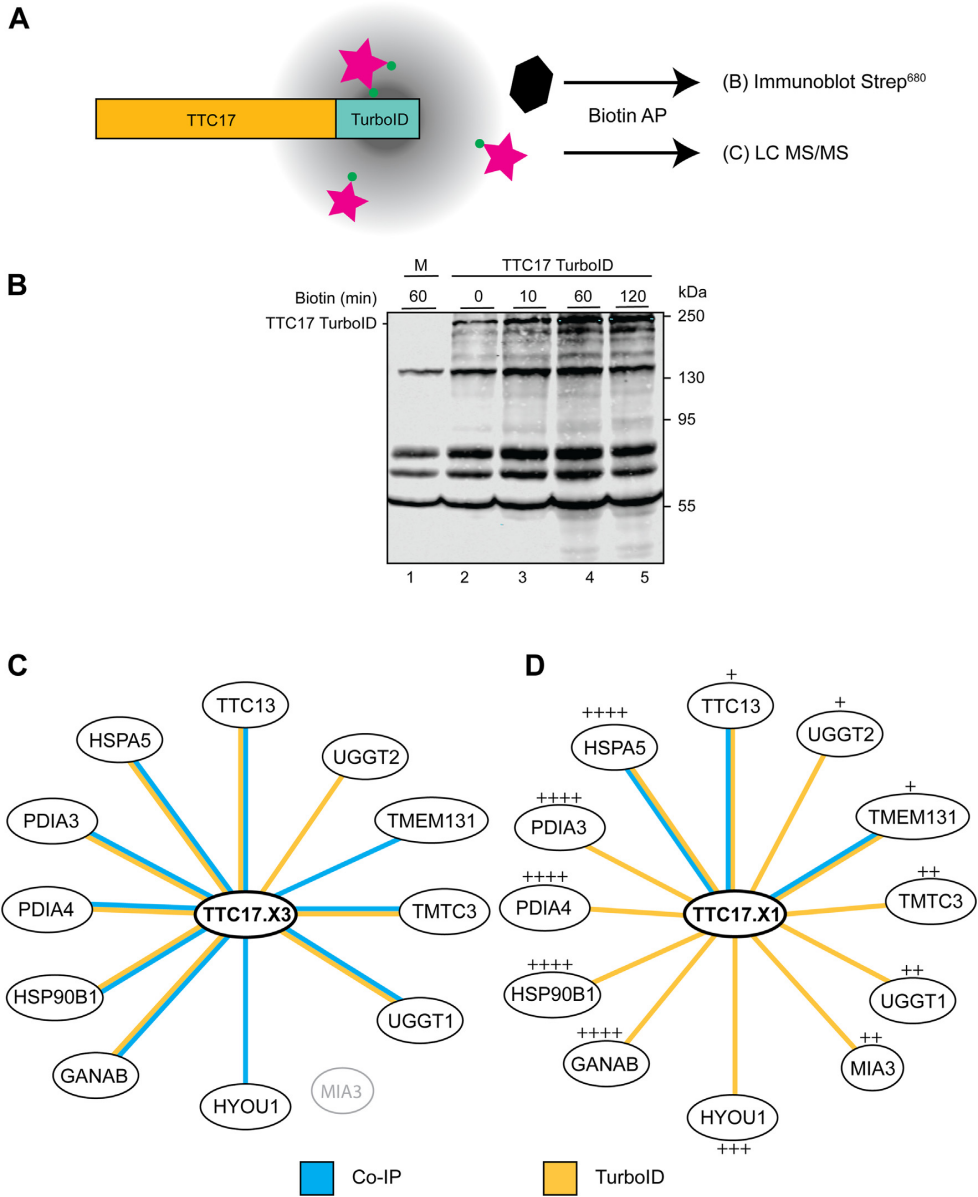
**TTC17 suborganellar localization determined *via* TurboID-mediated proximity labeling.***A*, a schematic representation of proximity labeling mediated by the TTC17 TurboID construct. Cells are transfected with the TTC17 TurboID copy DNA for 24 h, whereupon the transfected cells are incubated with 50 μM biotin for 1 h before quenching the reaction on ice. Cells were lysed and lysate was purified by biotin affinity purification *via* streptavidin beads. Substrates are prepared and sent for LC-MS/MS identification. *B*, a representative blot of proximity labeled samples from TTC17.X1 TurboID proximity labeling. HEK293T samples were transfected with TTC17 TurboID and after 24 h were incubated with 50 μM biotin for the indicated times. Samples were purified from cell lysate by biotin affinity purification and analyzed *via* SDS-PAGE. The blot was probed with streptavidin conjugated with a fluorophore. *C*, highly enriched proteins across the TTC17.X3 IP LC-MS/MS and TurboID proximity labeling experiments. The color of the lines connecting proteins to TTC17 center indicate experiment type, all are representative of three independent experiments. *D*, results for the IP LC-MS/MS and proximity labeling of TTC17.X1 represented as in (*C*). Protein expression levels from a quantitative MS study (1) in HeLa cells are denoted by: + (1–10 nM);++ (10–100 nM);+++ (100–1000 nM); and ++++ (>1000 nM). IP, immunoprecipitation; MS, mass spectrometry.

The TTC17 TurboID constructs were transiently transfected into *TTC17*^*−/−*^ HEK cells for 24-h prior to incubation with 50 μM biotin (1 h) for proximal protein biotinylation (Fig. 3, *A* and *B*, isoform X1 displayed). The time course selected for TurboID labeling was determined for saturation and labeling efficacy, with 1 h being chosen for saturation as TurboID has a lower efficiency for biotinylation in the ER (39). Biotinylated substrates from cell lysates were isolated by affinity purification and then identified *via* LC-MS/MS, with untransfected samples used to control background peptide identification.

The most enriched substrate for the TTC17.X3 TurboID proximity labeling was UDP-glucose:glycoprotein glucosyltransferase 1 (UGGT1), a key component of the lectin chaperone cycle in the ER (Fig. 3*C* and Table S3). Many of the secretory proteins enriched in the assay are ER chaperones or cochaperones verifying an ER localization for TTC17 and implying that TTC17 may have a role in protein maturation and quality control. Several other identified proteins are involved in the lectin chaperone cycle (UGGT2 and glucosidase IIα (*GANAB*)) or the HSP70/90 cycles (BiP and GRP94). Moreover, the recently characterized transmembrane and tetratricopeptide repeat–containing protein 3 was also identified, an ER resident O-mannosyltransferase (13).

The most enriched protein from the TTC17.X1 TurboID proximity labeling experiment is TANGO1 (also known as MIA3), along with other specialized secretory proteins. Several of the top proteins identified by proximity labeling to TTC17 had ERES localization. These proteins include TANGO1, UGGT1, and TMEM131 (Fig. 3*D*) (40, 41, 42, 43). Other low abundant proteins identified included ERdj5 and OS-9 that play roles in ERAD also expected to be found further into the ER (1, 44, 45). Together, the proximity labeling results verify an ER localization for TTC17 and are suggestive of a suborganellar localization at exit sites from the ER for vesicles headed to the Golgi (ERES) or retrotranslocation to cytoplasm for proteasomal degradation (ERAD).

We further looked to identify interaction partners of TTC17 by co-IP. A fusion construct of TTC17 followed by a C-terminal 3× FLAG tag was generated for both the X1 and the X3 isoforms of TTC17. This fusion construct was transiently transfected into *TTC17*^*−/−*^ HEK cells and interactors of TTC17 were isolated by pulldowns and identified by LC-MS/MS, using untransfected cells to control for background peptide identification. The most enriched substrate across three samples for TTC17.X3 was transmembrane protein 131 (TMEM131), a transmembrane trafficking chaperone involved in the proper secretion of collagen (42)(Fig. 3*C* and Table S3). Additionally, other ER resident proteins were highly enriched, namely GRP94, UGGT1, calnexin, and the ER localized TPR-containing protein TTC13. The TTC17.X1 IP LC-MS/MS also identified TMEM131 and TTC13 as highly enriched, similar to that of the X1 sample (Fig. 3*D*). Altogether, both the proximity labeling and co-IP results confirm ER localization, indicate that TTC17 may be acting at the ERES and identify a number of possible interactors.

### TTC17 is upregulated by ER stress

Proteins that reside within the ER are commonly important for maintaining protein homeostasis and both transcriptionally and translationally upregulated under stress (37). To assay *TTC17* transcriptional regulation under stress, HEK293A cells were subjected to various ER stressors, including tunicamycin (N-glycan synthesis inhibitor), thapsigargin (calcium dysregulation), DTT (reducing agent or redox stressor), brefeldin A (BFA, secretory trafficking inhibitor) and MG132 (proteasome inhibitor). RNA was harvested from cells and reverse transcribed to cDNA, followed by quantitative reverse transcription polymerase chain reaction with appropriate primers to monitor changes in transcript levels.

**Figure 4:**
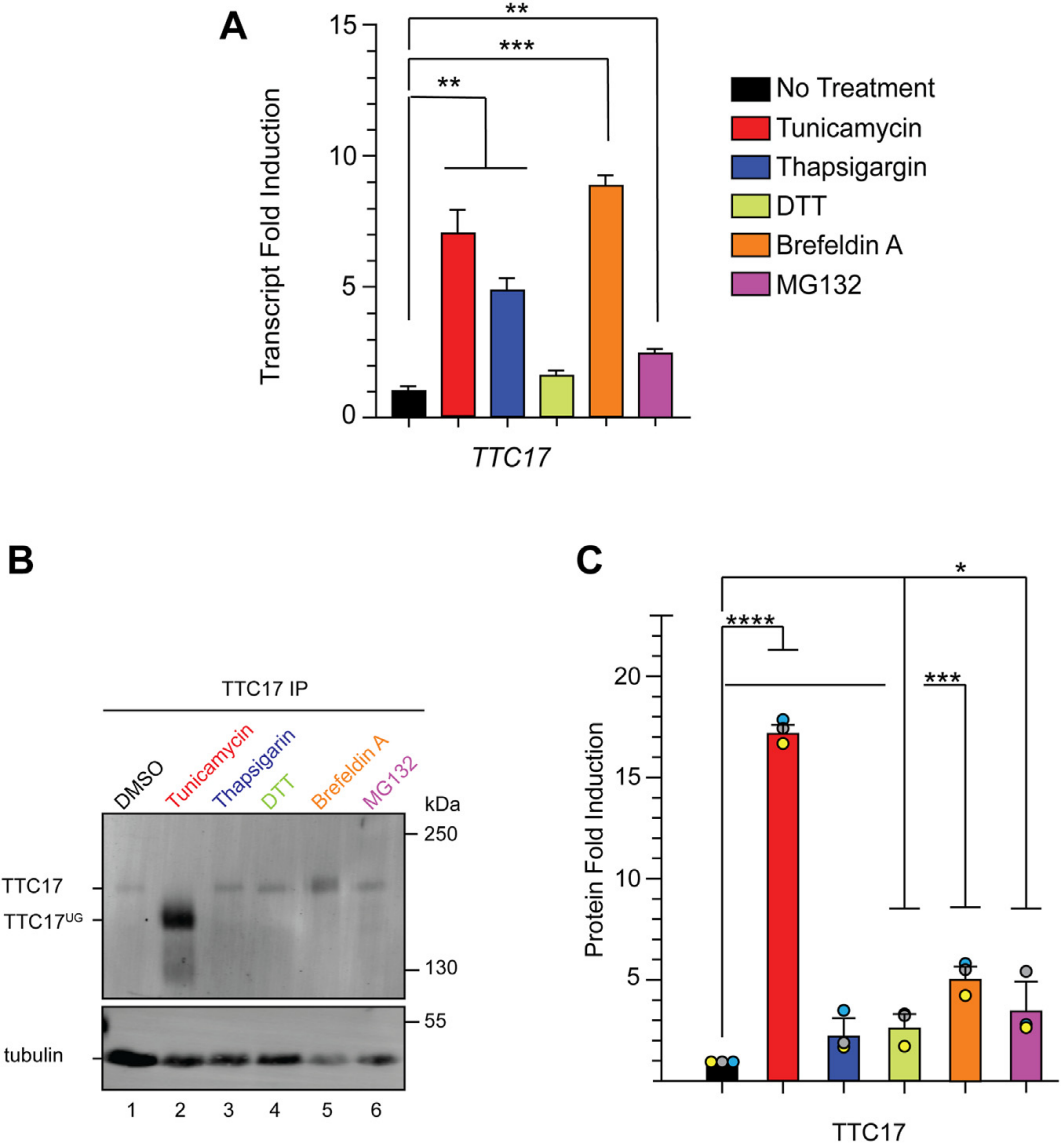
**TTC17 is upregulated under ER stress.***A*, HEK293A cells were treated with regular growth media or with 1 mg/ml tunicamycin (24 h), 3 μM thapsigargin (24 h), 2 mM DTT (2 h), 2.5 μg/ml brefeldin A (24 h), or 2.5 μM MG132 (24 h) prior to RNA purification. RNA was reverse transcribed to copy DNA, followed by quantitative reverse transcription polymerase chain reaction with appropriate primers, and changes in gene expression were calculated using β-actin as a reference. Statistical significance was determined using an unpaired *t* test. ∗, ∗∗, and ∗∗∗ indicated a *p*-value of less than 0.05, 0.01, and 0.001, respectively. Error bars represent SD from at least three independent experiments. *B*, HEK293T cells were treated as above for 24 h. Cells were lysed and TTC17 was purified *via* immunoprecipitation with TTC17 antisera and immunoblotting for TTC17. Samples were analyzed *via* SDS-PAGE. Quantification of three independent experiments shown in (*C*) with statistical significance determined as in (*A*). ER, endoplasmic reticulum.

*TTC17* transcript levels were increased most significantly with BFA (8.9-fold), tunicamycin (7.1-fold), and thapsigargin (4.9-fold) treatments (Fig. 4*A*). All stress conditions upregulated *TTC17* transcript expression when compared to the untreated sample, indicating *TTC17* expression is significantly upregulated by general ER stresses.

Upon unfolded protein response activation, general protein translation is inhibited to facilitate the trafficking of proteins through the secretory pathway or for degradation to alleviate the ER stress (37). Proteins with functions that serve to facilitate trafficking and stress reduction may be unaffected or upregulated translationally. As transcript levels do not always directly correlate with protein levels (46), TTC17 protein levels were analyzed under the same stress conditions as the transcript levels. HEK293T cells were subjected to the same range of ER stress conditions as above for 24 h before the cells were harvested. Cell lysates were immunoprecipitated with an endogenous TTC17 antibody and protein levels were quantified by immunoblotting for TTC17 or tubulin as a loading control.

TTC17 protein upregulation was most evident upon tunicamycin (18.1-fold) and BFA (6.9-fold) treatment (Fig. 4, *B* and *C*). The increased expression of TTC17 seen with tunicamycin exposure was not caused by an increase in the immunogenicity of unglycosylated TTC17 as PNGaseF treatment did not support an increase in TTC17 levels (Fig. 2*A*). Band broadening on the immunoblot under BFA stress was likely the result of complex N-glycans added to TTC17 when the Golgi collapsed into the ER upon treatment (47). Together, both TTC17 transcript and protein expression were upregulated by ER stress conditions, supporting a role for TTC17 in maintaining protein homeostasis in the early secretory pathway.

### TTC17 expression is cell type–specific

**Figure 5:**
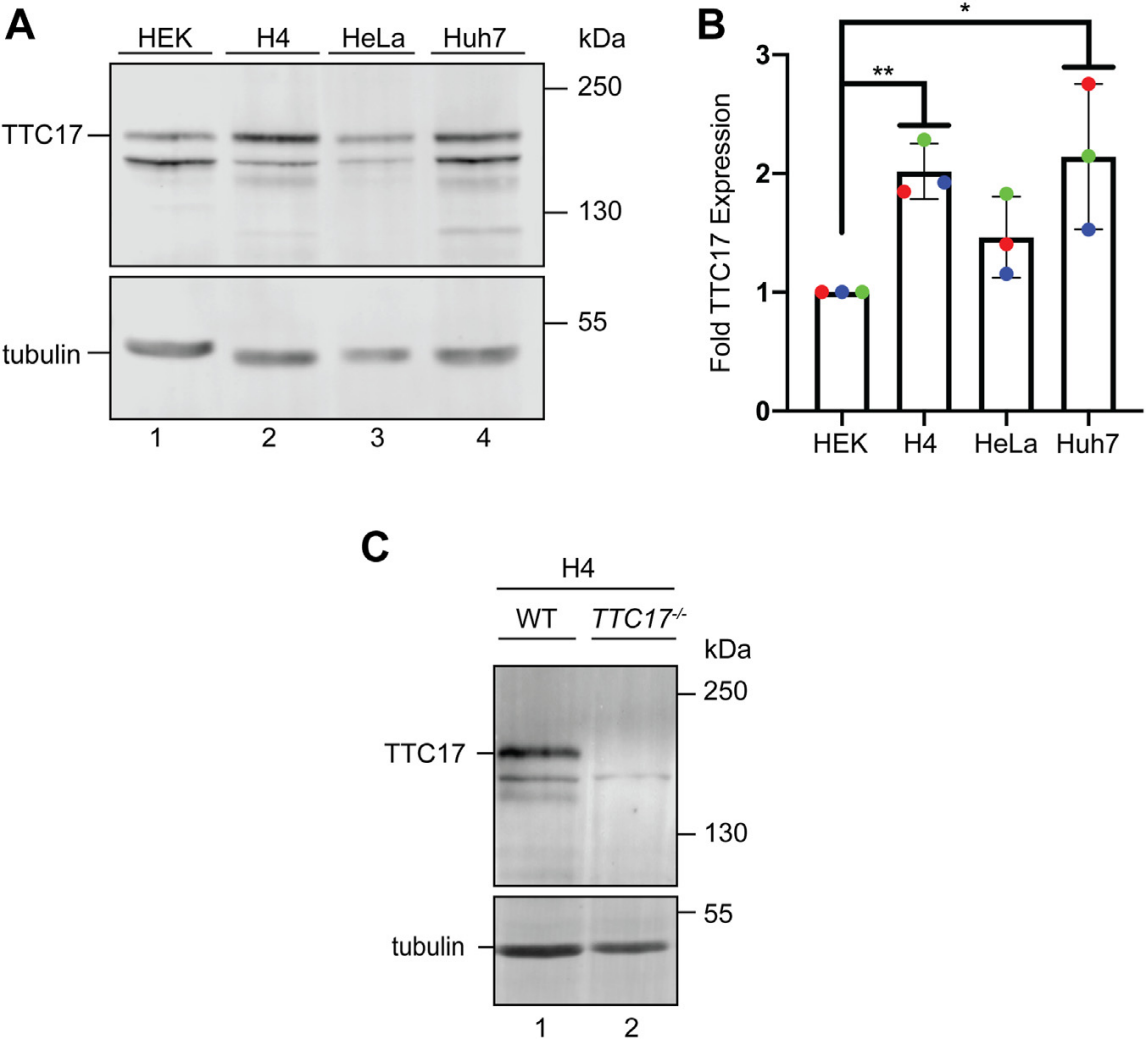
**TTC17 expression varies across cell lines.***A*, HEK, H4, HeLa, and Huh7 cells were harvested and lysates analyzed *via* immunoblotting against endogenous TTC17. *B*, quantification of replicates using beta tubulin for normalization across cell lines is displayed. *C*, H4 cells were used to generate a *TTC17*^*−/−*^ CRISPR-Cas9–edited cell line. Representative blots of the cell lines are shown with the indicated band absent in the knockout. Validation *via* immunoblot against endogenous TTC17.

Analysis of available transcript databases show that *TTC17* transcripts are expressed in a variety of different cell or tissue types at varying levels (Fig. S2). The level of TTC17 protein expression in several commonly used cell lines including HEK, H4 neuroglioma cells, HeLa, and Huh7 hepatocytes was determined. Cell lysates were analyzed by immunoblotting with TTC17 antisera using tubulin as a loading control to normalize across the cell lines (Fig. 5*A*). TTC17 expression was found to be the highest in the H4 and Huh7 cell lines when compared to the HEK and HeLa cells.

To further explore properties and possible roles for TTC17 in a cell line where *TTC17* was expressed at a high level, a CRISPR-edited H4 *TTC17* KO cell line was generated (Fig. 5*C*), in addition to the previously shown *TTC17*^*−/−*^ HEK cell line (Fig. 1*C*). The ∼170-kDa protein band that corresponds to TTC17 is absent in the H4 *TTC17*^*−/−*^ cell line. The HEK and H4 *TTC17*^*−/−*^ cell lines will be used below to explore the roles for TTC17 in protein trafficking in the secretory pathway.

### The absence of TTC17 leads to trafficking defects

**Figure 6:**
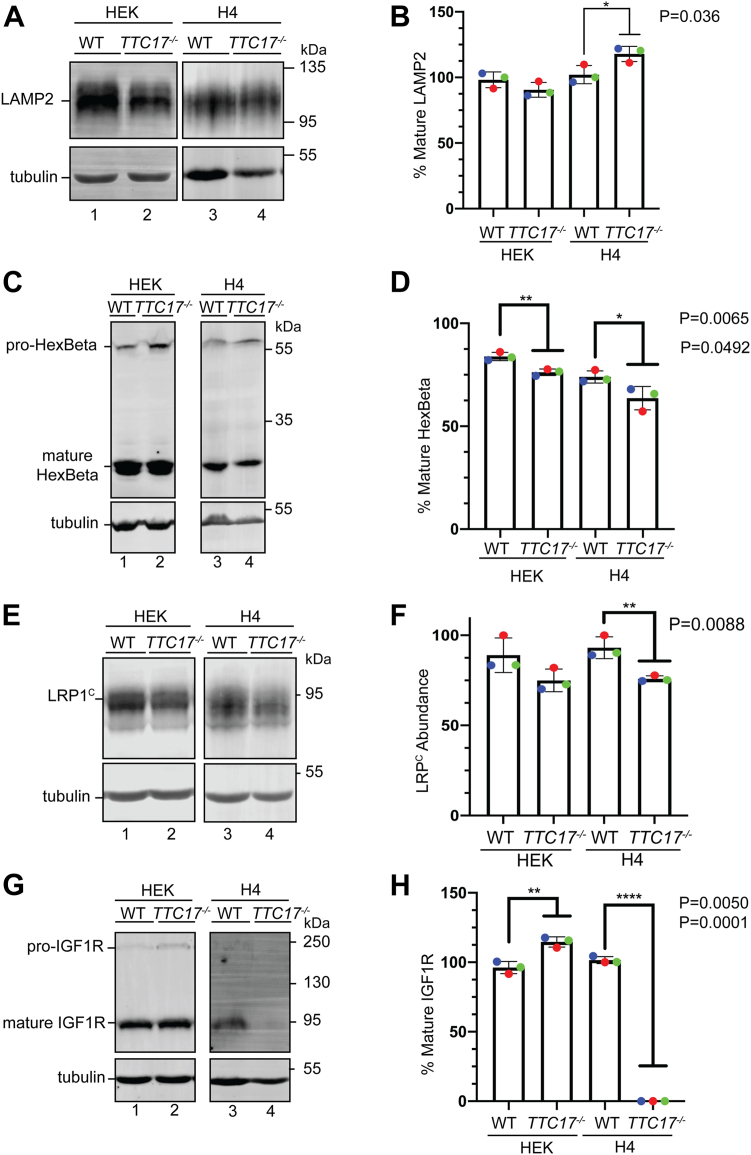
**TTC17**^**−/−**^**exhibits trafficking defects for a variety of secretory pathway clients.***A*, designated cell lines were lysed and analyzed *via* immunoblot against LAMP2, expression normalized to beta tubulin across the cell lines. *B*, quantification of three independent experiments is displayed. *C*, processing/trafficking β-hexosaminidase subunit β assayed as described in (*A*) with quantification (*D*). *E*, trafficking of LRP1 assayed as previously described (*A*) with quantification (*F*). LRP1^c^ denotes the processed C-terminal membrane portion of LRP1. *G*, trafficking of IGF1R as previously described (*A*) with quantification (*H*). IGF1R, insulin-like growth factor type 1 receptor; LRP1, lipoprotein receptor–related protein 1.

The identification of clients possibly affected by the knockout of *TTC17* in HEK and H4 cell lines was initially probed in a targeted manner by assaying a variety of proteins for which their trafficking could be followed upon exiting the ER to the lysosome or the cell surface. First, LAMP2 is a highly glycosylated integral membrane lysosomal protein indicated to be aberrantly glycosylated when TTC17 was knocked down using siRNA in HeLa cells (26). In both the HEK and H4 *TTC17*^*−/−*^ cell lines, LAMP2 was not aberrantly glycosylated. However, LAMP2 expression was slightly upregulated (15.8%) in the H4 *TTC17*^*−/−*^*,* while no significant difference was observed for expression in HEK cells (Fig. 6, *A* and *B*).

Next, the processing and trafficking of a soluble lysosomal protein β-hexosaminidase subunit β (HexB) was analyzed. HexB is processed into three disulfide bound chains in the lysosome (48). In both the H4 and HEK cells, the percent mature HexB minimally decreased, indicating a subtle increase in relative immature HexB in both cell types (Fig. 6, *C* and *D*).

To assay further trafficking aberration beyond lysosomal targeted proteins, low-density lipoprotein receptor–related protein 1 (LRP1) processing and trafficking to the plasma membrane was interrogated. LRP1 is a large (∼600-kDa) type I integral membrane protein that is composed of two subunits after processing by furin in the *trans*-Golgi: a 515-kDa extracellular subunit and an 85-kDa membrane bound subunit (LRP1^c^) (49, 50). In both the HEK and H4 cells, the expression of LRP1^C^ was reduced by 14.1 and 17.2% in the *TTC17*^*−/−*^ compared to WT cells, respectively, suggestive of a minimal role for TTC17 in the efficient trafficking and processing of LRP1 (Fig. 6, *E* and *F*).

Lastly, the trafficking of insulin-like growth factor type 1 receptor (IGF1R), a type I membrane protein that undergoes furin-like cleavage and trafficking to the plasma membrane was followed (51, 52). There was an 18.4% increase in the expression of mature IGF1R in the HEK *TTC17*^*−/−*^ cells as compared to WT HEK cells (Fig. 6, *G* and *H*). Strikingly, mature IGF1R expression in the H4 *TTC17*^*−/−*^ cells was not detected by immunoblotting (Fig. 6, *G* and *H*). *IGF1R* was not perturbed transcriptionally, as there was no significant difference between H4 WT and *TTC17*^*−/−*^ IGF1R transcription *via* RT-qPCR (Fig. S3*A*). IGF1R translation occurs similarly in the H4 WT and *TTC17*^*−/−*^ cells as shown with a 30 min and 60 min [^35^S]-Met/Cys radioactive pulse, followed by an IGF1R IP (Fig. S3*B*). Thus, the aberrant expression of IGF1R in the H4 *TTC17*^*−/−*^ cells appears to be posttranslational, however inhibiting the proteasome *via* incubation for a range of times with MG132 had little effect on IGFR1 expression in *TTC17*^*−/−*^ H4 cells (Fig. S3*C*).

Taken together, these results indicate that TTC17 has varying effects on the maturation and trafficking of proteins within the secretory pathway. In the absence of TTC17, secretory pathway clients with a wide variety of characteristics are differentially affected. These results were cell type–specific with the H4 neuroglioma cells that express higher levels of TTC17 exhibiting the most significant trafficking defects especially as observed for IGFR1.

### Global analysis of trafficking in TTC17^−/−^ cells

To characterize more extensively the changes in trafficking due to the absence of TTC17, a secretory pathway wide quantitative proteomics approach was taken. In brief, the secretory pathway or endomembranes were fractionated and enriched from cell homogenates. The enriched endomembrane and plasma membrane microsomes were then prepared for quantitative tandem mass tagging (TMT) MS to compare the protein content of WT and *TTC17*^*−/−*^ H4 cell lines. This permits the characterization of the effects of the knockout in an unbiased and secretory pathway specific manner.

**Figure 7:**
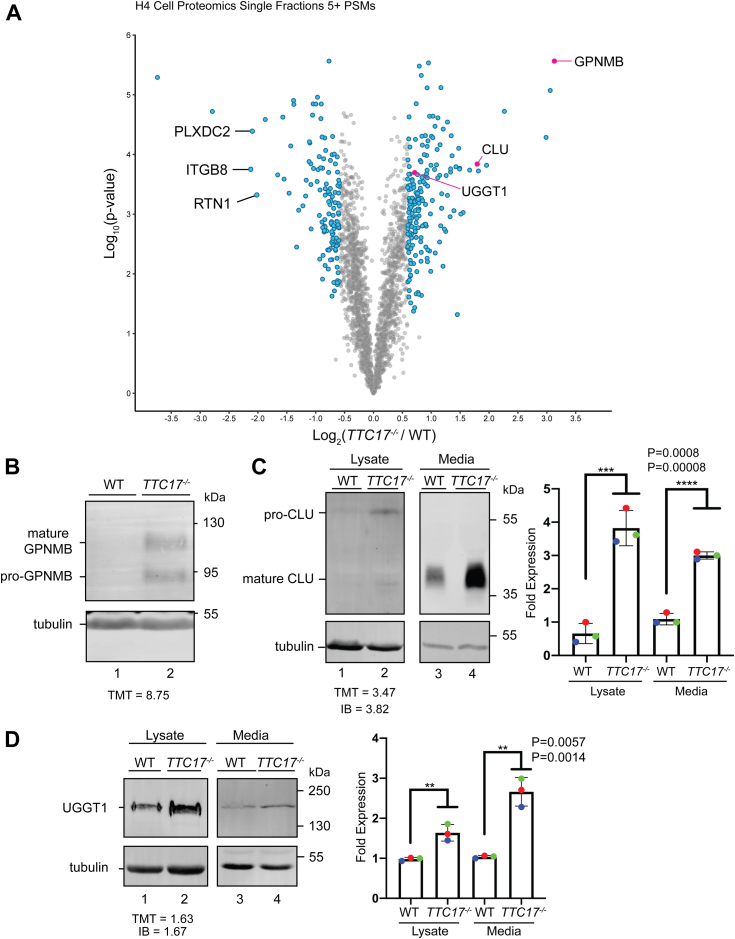
**TTC17**^**−/−**^**in H4 cells affects a wide array of secretory proteins.***A*, proteins were analyzed by dividing the quantification of the TMT label in the *TTC17*^*−/−*^cell line for each protein by that of the associated quantification of the TMT label in the WT cell line. Data is representative of three independent experiments. Highlighted (*blue*) points represent both *p*-value <0.05 and >0.59 or < −0.59 quantification enrichment. Highlighted (*magenta*) points represent substrates chosen for further validation. *B*, WT and *TTC17*^*−/−*^ H4 cells cell lysates were analyzed *via* immunoblotting against endogenous GPNMB. A representative blot of N = 3 is shown. *C*, intracellular clusterin (CLU) assayed as in (*B*). Media samples were taken and analyzed similarly with representative blot on the *left* and quantification of three independent experiments on the *right*. *D*, UGGT1 analyzed as described in (*C*). GPNMB, glycoprotein nonmetastatic melanoma protein B; TMT, tandem mass tag; UGGT, UDP-glucose:glycoprotein glucosyltransferase.

Using this quantitative proteomics framework, ∼2600 proteins were identified and quantified across three biological replicates for both WT and *TTC17*^*−/−*^ H4 cells (Fig. 7*A*). Further, over 300 different proteins were highlighted as both significant and highly upregulated or downregulated in H4 *TTC17*^*−/−*^ cells when compared to WT H4 cells. The identified proteins cover a wide range of secretory pathway client proteins. As over 300 proteins were identified as top hits, we chose to validate a selection across a range of enrichments, topologies and localizations (Fig. 7*A*, magenta dots).

The transmembrane glycoprotein nonmetastatic melanoma protein B (GPNMB) was found to be the most highly enriched protein in the*TTC17*^*−/−*^ H4 cells (Figs. 7*A*, 8.8-fold). GPNMB is a transmembrane protein, which traffics to the plasma membrane and has been shown to interact with α-synuclein, increasing the progression of Parkinson’s disease (53). GPNMB was confirmed to be upregulated in *TTC17*^*−/−*^ H4 cells as compared to WT cells, although quantification was not possible as GPNMB was undetectable in WT H4 cells (Fig. 7*B*).

TMT proteomic analysis and immunoblotting indicated enrichment of the secreted glycoprotein clusterin (CLU) levels in the absence of TTC17. CLU was enriched both intracellularly (TMT 3.5-fold and immunoblot 3.8-fold) and extracellularly (3-fold) (Fig. 7*C*).

Lastly, UGGT1 enrichment in *TTC17*^*−/−*^ cells was tested as it was also previously identified by proximity labeling and co-IPs (Fig. 3*C*). UGGT1 was enriched by 1.67-fold *via* immunoblotting in the *TTC17*^*−/−*^ H4 cells as compared to WT cells, which closely matched the enrichment of 1.63-fold determined by quantitative proteomics (Fig. 7*D*). Additionally, extracellular UGGT1 was also enriched by 2.7-fold *via* immunoblotting in the *TTC17*^*−/−*^ H4 cells, indicating that more UGGT1 leaked out of the cell in the absence of TTC17 (Fig. 7*D*).

Altogether, these results indicate that TTC17 affects the maturation and trafficking of a wide variety of proteins in the secretory pathway. Furthermore, they confirm the cell type specificity with the H4 neuroglioma cells as previously asserted, as the HEK cells experienced little change in the absence of TTC17 (Fig. S4). Whereas the absence of TTC17 in the H4 neuroglioma significantly altered the expression of over 300 proteins.

## Discussion

Proper protein maturation, trafficking, and secretion are integral to cellular homeostasis. A wide variety of ER quality control proteins have been identified that serve to maintain protein homeostasis (54). Here, we characterized the impact the novel resident ER protein TTC17 has on secretory protein trafficking. Our identification of TTC17 started as an unbiased *in silico* screen utilizing the TPR motif and potential ER localization signal sequences as the determinants for the basis of possibly identify new ER localized quality control factors.

As there are multiple isoforms of TTC17 displayed in UniProt, the isoform of TTC17 expressed in human cell lines was delineated (Fig. 1, *B* and *D*). Transcriptionally and translationally, TTC17 isoform 3 (TTC17.X3) is the dominant isoform expressed in H4 and HEK cells. A spliced isoform missing 57 amino acids (TTC17.X1) also appears to be expressed but at a much lower level. The impact of differential expression of TTC17 isoforms has yet to be investigated; however, they may serve to dynamically modulate different secretory stresses and quality control mechanisms.

Previous studies described TTC17 as either a cytoskeletal interacting protein or a Golgi localized secretory trafficking factor (9, 26). In light of this inconsistency in published studies, we illuminated the cellular localization of TTC17 using a number of approaches. SignalP6.0 and Deeploc1.0 algorithms predicted a secretory pathway or more specifically an ER localization for TTC17 (22, 55). Secretory pathway targeting was confirmed by the N-glycosylation of TTC17 observed using a PNGaseF sensitive assay (Fig. 2*A*). The absence of TTC17 from the cell media was indicative of its residence within the cellular secretory/endocytic pathway. EndoH sensitivity mirrored that of PNGaseF treatment suggestive of TTC17 possessing high mannose or ER glycoforms. Interestingly, the size shift of TTC17 upon deglycosylation and IP LC-MS/MS both are consistent with the majority of the glycosylation sites on TTC17 being populated, making TTC17 one of the most glycosylated soluble resident ER proteins (Fig. 2*F*). A number of the N-glycans are placed within the TPR regions, as also observed with SEL1L (56). The necessity of these glycans for substrate recognition on SEL1L and TTC17 is a subject for further study.

ER residency was confirmed by confocal immunofluorescence microscopy of overexpressed TTC17 (Fig. 2, *B* and *C*). Proximity labeling *via* TurboID also supported ER localization and was suggestive of ER subdomain concentration to the peripheral ER (Fig. 3*C*). Specifically, proximity labeling identified specialized collagen trafficking machinery (TANGO1) along with members of the lectin chaperone quality control system (UGGT1/2 and GlsIIα) and other maturation machinery. The identification of the UGGTs may be a result of reglucosylation of TTC17, however the lectin chaperones calreticulin and calnexin are not as highly enriched across the MS results and UGGT1 has been localized to ERES or pre-Golgi sites by immunoelectron microscopy (43). Most strikingly was the identification of TANGO1 and TMEM131, both proteins are expressed in low abundance and are directly involved in the specialized trafficking of collagen at the ERES (1, 40, 42). The identification of TMEM131 with both isoforms of TTC17 is especially intriguing, as extensive characterization of TMEM131 in mammalian cells has yet to be conducted (42). This finding leads to the possibility that TTC17 may be functioning at ERES.

ER quality control proteins are often stress regulated (37, 57). *TTC17* was transcriptionally upregulated significantly across various ER stress conditions, with tunicamycin (glycan synthesis inhibition) and brefeldin A (secretory trafficking inhibition) both being the most significant upregulators of stress conditions (Fig. 4*A*). Protein expression of TTC17 was also upregulated similarly by all stress conditions; however tunicamycin induced upregulation most significantly with an 18.1-fold increase (Fig. 4, *B* and *C*). Conclusively, TTC17 is both transcriptionally and translationally upregulated by ER stress, which is indicative of TTC17 having an important role in the ER stress response. Given the previous data illustrating a possible role as a general trafficking factor, TTC17 may facilitate amelioration of ER stress by moderating protein maturation within the ER or mediate the trafficking of secretory proteins further down the secretory pathway (26).

A previous study identified TTC17 as a possible trafficking factor using siRNA-mediated knockdown of TTC17 in HeLa cells (26). Our work assayed proteins affected by the knockout of *TTC17* in two cell lines. From the targeted approach using immunoblots of whole-cell lysates (WCLs), several potential substrates were identified and characterized, with IGF1R being the most strikingly affected protein (Fig. 6, *G* and *H*). Further, this phenomenon was not observed transcriptionally, nor *via* an abrogation in translation in the *TTC17*^*−/−*^ H4 cells (Fig. S3, *A* and *B*). Thus, the loss of mature IGF1R in the *TTC17*^*−/−*^ H4 cells seems to be a posttranslational degradation mediated process, though inhibition of the proteasome utilizing the inhibitor MG132 did not have noticeable affect (Fig. S3*C*). What specifically is mediating the posttranslational loss of IGF1R in the *TTC17*^*−/−*^ remains to be seen and is currently under investigation. However, the stark results regarding the maturation of IGF1R specifically in the H4 *TTC17*^*−/−*^ cells highlights the possibility of other substrates being affected throughout the secretory pathway proteome.

With the success of targeted substrate identification, a secretory proteome wide quantitative method for identifying substrates was developed. Utilizing an unbiased quantitative proteomics framework, the absence of *TTC17 via* CRISPR/Cas9-mediated knockout resulted in the identification of over 300 proteins, which had substantial altered enrichment in the *TTC17*^*−/−*^ as compared to WT H4 neuroglioma cells (Fig. 7*A*). The identified proteins ranged broadly in function, localization, solubility, and size. The most upregulated protein identified in the *TTC17*^*−/−*^ H4 cells was GPNMB, a transmembrane glycoprotein implicated in the progression of various neurological diseases (53). This connection with neurological disease progression was shared with another identified upregulated protein, CLU, a secreted glycoprotein involved in interaction with tau and α-synuclein (58).

Intriguingly, the identification of UGGT1 as an intracellularly upregulated protein in the *TTC17*^*−/−*^ H4 cells *via* TMT-MS was confirmed and the leakage of UGGT1 was additionally found to be upregulated in *TTC17*^*−/−*^ H4 cells when compared to WT (Fig. 7, *A* and *D*). The localization of UGGT1 to peripheral ER and sites of ER trafficking was previously shown *via* immunogold labeling, however the mechanism for specific localization and further ER retention of this soluble chaperone is currently unknown (43).

Many ER proteins contain the canonical ER retrieval motif Lys-Asp-Glu-Leu (KDEL) at the C terminus, a motif which binds to the KDEL receptor (KDELR) in the Golgi. The KDELR cycles between the ER and the *cis-*Golgi, binding and releasing proteins *via* the KDEL motif in a pH-dependent manner in order to retrieve proteins that leak out of the ER (59, 60). UGGT1 does not contain a KDEL motif, instead it has a Lys-Arg-Glu-Glu-Leu (KREEL) C-terminus motif, and as such the mechanism for the retrieval of UGGT1 is currently unknown. Furthermore, there is very little knowledge about the mechanism of retention for ER proteins before capture and retrieval by the KDELR from the Golgi. Thus, with UGGT1 leakage increased in the absence of TTC17, could TTC17 be responsible for the retention of UGGT1 specifically or perhaps does TTC17 have a wider and less specific role regarding retention of ER resident proteins?

The size of the respective proteome from yeast to metazoans has more than tripled and predictively the proteins targeted to the secretory pathway in each organism have increased by even more (61). Along this evolutionary ascent, various specialized secretory proteins arose and are utilized in higher metazoans not only were additional protein chaperones developed but secretory protein complexes arose. For example, in the human ER N-linked glycans are added both cotranslationally and posttranslationally *via* a second translocon-associated OST not present in the lower eukaryotes that works co-translational before folding commences (31, 62). Basic Local Alignment Search Tool analysis indicated that TTC17 is conserved in chordata but is not present in lower eukaryotes like *Drosophila melanogaster*, *Caenorhabditis elegans*, or yeast— corresponding to the increase in proteomic size and secretory complexity stated above. TTC17 may be a soluble trafficking chaperone or adaptor that modulates proper substrate trafficking and resident retention at ERESs that arose due to the inherent increase in complexity of the secretory pathway in higher metazoans. H4 cells express 4-fold the level of BiP compared to HEK cells, suggestive of the neuroglioma cells of possibly having a more robust secretory pathway capacity than the kidney cells (Fig. S5). Further studies are needed to fully elucidate the specific mechanistic role that TTC17 has in the ER and if its function is a specific targeted interaction or a wider and more promiscuous chaperone function across a wide variety of substrates and cell types.

## Experimental procedures

### Cell culture

HEK293 cells, Huh7, and HeLa were acquired from American type tissue collection and cultured in Dulbecco's modified Eagle's medium (DMEM) (Gibco) supplemented with certified 10% fetal bovine serum (FBS, Gibco) at 37 °C with 5% CO_2_. HEK293-EBNA1-6E (HEK) cells were employed and used as the parental line to create one of the CRISPR/Cas9 edited *TTC17*^*−/−*^ cell lines (63). HEK cells were cultured in DMEM supplemented with certified 10% FBS at 37 °C with 5% CO_2_. H4 cells were also employed and used as a parental line to create a *TTC17*^*−/−*^ cell line. H4 cells were cultured in DMEM supplemented with 10% FBS at 37 °C with 5% CO_2_. Cells were tested for the presence of *mycoplasma* using a universal *mycoplasma* detection kit (American type tissue collection, Cat # 30-012K).

### TTC17 sequencing

HEK and H4 cells were plated and grown for 48 h prior to harvest for mRNA extraction utilizing PureLink RNA Mini Kit (Thermo Fisher Scientific). mRNA was extracted as per the manufactures protocol. Purified mRNA (1 μg) was reverse transcribed to generate cDNA using the Protoscript II Reverse Transcriptase kit (New England Biolabs, (NEB)). TTC17 specific cDNA was further amplified using primers specific for 5′ and 3′ ends of the cDNA for *TTC17*. A PCR product was then cloned into a backbone vector and sequenced by Sanger sequencing.

### CRISPR/Cas9 KO generation

HEK *TTC17*^*−/−*^ and H4 *TTC17*^*−/−*^ were generated *via* CRISPR/Cas9 using plasmid PX459 from Addgene with the targeting guide RNA (gRNA) 5′ - CACGCACTGGGTCGTCACGG - 3′ (64). KO lines were generated by transfecting the HEK/H4 cells at 70% confluency in a 10-cm plate with 8 μg of the gRNA CRISPR/Cas9 plasmid, using 2.5:1 PEI:DNA and 1:1 GeneXPlus:DNA, respectively. Cells were grown for 24 h before the media was replaced with media containing puromycin as a selective pressure. The cells were then grown under puromycin selection for 5 days. Cells were then trypsinized and plated into 96-well plates in a limiting dilution to isolate single-cell colonies. Knockouts were confirmed by immunoblotting and staining for TTC17, along with genomic DNA extraction and amplicon sequencing around the gRNA target sequence. Growth and gross cellular morphology were unchanged upon *TTC17* knockout.

### Glycosidase assay

3.5 × 10^6^ HEK cells were plated in a 10-cm plate and were either allowed to grow for 48 h or transfected after 24 h with the indicated construct. Cells were lysed in 900 μl MNT buffer (20 mM 2-(N-morpholino) ethanesulfonic acid, 100 mM NaCl, 30 mM Tris–HCl, 0.5% Triton X-100, pH 7.5) with protease inhibitor cocktail (Thermo Fisher Scientific) and 20 mM n-ethylmaleimide. Proteins were then immunoprecipitated with the indicated antibody overnight at 4 °C. Beads were washed twice with MNT, whereupon samples were incubated with PNGaseF or EndoH following the manufacturer’s instructions (New England Biolabs). Samples were diluted 1:1 with reducing sample buffer and analyzed with immunoblotting.

### Immunofluorescence confocal microscopy

1 × 10^5^ COS7 cells were added to a 12-well plate containing a glass coverslip and cultured for 24 h in DMEM completed with 10% FBS prior to transfection. *TTC17*^*FLAG*^cDNA was transfected according to methods described earlier and cultured for 24 h. The next day, the media was removed, and the coverslips were washed with once with PBS before being fixed with 4% paraformaldehyde in PBS for 15 min, followed by permeabilization with 0.1% Triton X-100 for 15 min at 25 °C. After permeabilization, blocking buffer (10% FBS in PBS) was added to each well for 30 min. The blocking buffer was removed and the slides were then washed three times with PBS and stained with an α-giantin (Golgi, 1:250) (Abcam, ab24586), α-KDEL (ER, 1:250) (Invitrogen, PA1-013), or α-FLAG antibody (TTC17, 1:100) (Sigma, F1804) for 2 h. After the primary staining, the cells were washed with PBS before incubating with an α-mouse Alexa Flour 594 (Invitrogen, A21203) and α-rabbit Alexa Flour 488 (1:500) (Invitrogen, A21206) for 1 h. Slides were again washed and mounted onto cover slips with VectaShield (Vector Laboratories). Images were obtained at the UMass Amherst Nikon Center of Excellence Core Facility (RRID: SCR_021148) using an A2R25 Nikon A1 Resonant Scanning Confocal. All images were acquired with a Plan Fluor 40× Oil DIC H N2 lens with a numerical aperture 1.3 and a refractive index of 1.515. Images were captured using a Fusion camera with a 200 ms exposure and processed using NIS-Elements AR. Individual cells were analyzed using a Bezier region of interest selection and Pearson’s correlation coefficients were obtained using NIS-Elements AR. All statistical analyses were completed using GraphPad Prism v9.

### Trypsin sensitivity assay

Samples were processed as previously described (13). Cells were grown for 24 h before transfection with TTC17 S-tag cDNA or left untreated. Twenty-four hours posttransfection the cells were pulse labeled for 1 h with EasyTag Express^35^S Protein Labeling Mix [^35^S]-Cys/Met (PerkinElmer). After 1 h of pulse labeling, the cells were washed 2× with PBS on ice. Cells were resuspended in cold homogenization buffer (10 mM Hepes, pH 7.4, 10 mM KCl, 1.5 mM MgCl, 5 mM sodium EDTA, 5 mM sodium EGTA, and 0.25 M sucrose) and passed through a 25-gauge needle 20×. All subsequent steps were conducted at 4 °C. The homogenate was centrifuged at 1000*g* for 10 min to pellet the nuclear fraction. The remaining supernatant was centrifuged at 45,000 rpm in a Beckman rotor (TLA 120.2) for 10 min to separate the cytosol (supernatant) from the endomembrane compartments (microsomes). The microsomes were resuspended in homogenization buffer containing 0.1 M NaCl and 10 μg trypsin, with or without Triton X-100 added to a final concentration of 0.1%. After incubation at 27 °C for 15 min, the reaction was quenched with 100 μg soybean trypsin inhibitor. Reducing sample buffer was added and analyzed *via* autoradiography.

### Alkaline extraction

Alkaline extraction was performed as previously described (13). Briefly, cells were resuspended in ice-cold homogenization buffer (20 mM Hepes, 5 mM KCl, 120 mM NaCl, 1 mM EDTA, and 0.3 M sucrose, pH 7.5) and passed through a 25-gauge needle 20×. All subsequent steps were conducted at 4 °C. A sample of the homogenate was taken as a whole-cell lysate sample. The homogenate was centrifuged at 1000*g* for 10 min to pellet the nuclear fraction (N). The supernatant was removed and centrifuged at 45,000 rpm in a Beckman rotor (TLA 120.2) for 10 min to separate the cytosolic fraction (C) from the TM pellet. The endomembrane fraction was resuspended in homogenization buffer and incubated with 0.1 M Na_2_CO_3_ (pH 11.5) for 30 min on ice. After incubation, the sample was underlaid with a sucrose cushion (50 mM triethanolamine, 0.3 M sucrose, pH 7.5). This sample was then centrifuged at 65,000 rpm for 20 min through the cushion to separate soluble proteins from membrane proteins in the supernatant and pellet, respectively. The pH was adjusted in the alkaline extracted sample with 1 M Tris–HCl, pH 7.5. The fractions were then analyzed *via* SDS-PAGE, followed by immunoblotting.

### Proximity labeling

3.5 × 10^6^ HEK cells were seeded in a 10-cm plate and grown for 24 h until they were transfected with TTC17 TurboID cDNA. Twenty four hours posttransfection, the cells were incubated with 50 μM biotin for 1 h. After the labeling, the cells were immediately put on ice and washed 4× with PBS to remove any excess biotin and halt the promiscuous biotin labeling. Cells were lysed with radio-immunoprecipitation assay buffer (150 mM NaCl, 50 mM Tris–HCl, 0.1% SDS, 0.5% sodium deoxycholate, 1% Triton X-100, pH 8) and sonicated for 20 s. Cells were then affinity purified with streptavidin-agarose (Thermo Fisher Scientific) and prepared for LC-MS/MS.

### IP and preparation for LC-MS/MS

One 10 cm plate was seeded with 3.5 million HEK cells and grown for 24 h prior to transfection with TTC17^FLAG^ cDNA. Cells lysed 24 h posttransfection in 900 μl MNT buffer with protease inhibitor cocktail (Thermo Fisher Scientific). Proteins were then immunoprecipitated with the FLAG-M2 antibody overnight at 4 °C. Beads were washed twice with MNT before incubation with 100 μl of elution buffer (200 mM glycine, pH 11) while shaking for 5 min. Beads were spun down at 800*g* for 10 min, and the supernant was transferred to a new microcentrifuge tube. The elution was repeated and the supernatants pooled for a total of 200 μl.

Eluate was then reduced with 30 mM DTT for 1 h, followed by 30 min incubation with 100 mM iodoacetamide in the dark. After alkylation, the sample was then precipitated with five volumes of acetone overnight at −20 °C. The precipitate was centrifuged at 20,000*g* for 10 min at 4 °C to pellet the protein sample. The supernatant was aspirated and discarded, while the pellet was resuspended in 400 μl of 15 mM triethylammonium bicarbonate (Thermo Fisher Scientific) and sonicated to fully suspend the pellet. The sample was incubated with 4 μg of trypsin/Lys-C (Promega) overnight at 37 °C. After proteolysis, the sample was treated at 95 °C to inactivate the proteases, and then the sample was treated with 2 μl of PNGaseF (NEB) overnight at 37 °C. After endoglycosidase treatment, peptides were quantified with the Pierce Quantitative Colorimetric Peptide Kit (Thermo Fisher Scientific).

### LC-MS/MS acquisition

LC-MS analysis was performed using an Easy-nLC 1000 nanoLC chromatography system interfaced to an Orbitrap Fusion mass spectrometer (Thermo Fisher Scientific). Samples were preconcentrated and desalted on to a C18 trap column prior to separation over a 90-min gradient from 0% to 40% mobile phase B (A: 0.1% formic acid in water, B: 0.1% formic acid in acetonitrile) at 300 nl/min flow rate with a 75 μm × 15 cm PepMap rapid separation liquid chromatography column (Thermo Fisher Scientific). MS parameters were as follows: ion spray voltage 2000V, survey scan MS1 120 k resolution with a 2 s cycle time, interleaved with data-dependent ion trap MS/MS of highest intensity ions with higher collisional dissociation at 30% normalized collision energy.

### ER stress treatments

HEK293A cells were treated with regular growth media or DTT (2 mM) for 2 h or tunicamycin (1 μg/ml), thapsigargin (3 μM), brefeldin A (2.5 μg/ml), and MG132 (2.5 μM) for 24 h prior to RNA isolation with the RNAeasy Mini Kit (Qiagen). Purified RNA (1 μg) was reverse transcribed into cDNA using the Protoscript II Reverse Transcriptase kit (NEB). Quantitative reverse transcription polymerase chain reactions were performed in 20 μl reactions using the FastStart universal SYBR Green master (Rox) kit (Roche Diagnostics) according to manufacturer’s instructions. Quantification was determined compared using beta actin as the reference gene (65). Statistical analysis of the data was calculated between treatment groups.

For TTC17 protein level determination, HEK293T cells were treated as described above with the ER stress agents accepted DTT treatment was for 24 h. Cells were lysed in MNT buffer. Cell lysates were split 90% for IP with TTC17 antibody and 10% of the lysate taken as WCL and precipitated with acetone for loading control normalization. Samples were then analyzed *via* immunoblot for TTC17.

### Secretory pathway TMT-MS sample preparation

One 10 cm plate was seeded with 2 million (H4) or 3.5 million (HEK) cells and allowed to grow for 48 h. Prior to homogenization, the media was aspirated and the cells were washed twice with PBS on ice. Cells were resuspended in 1 ml of homogenization buffer (20 mM Hepes, 5 mM KCl, 120 mM NaCl, 1 mM EDTA, and 0.3 M sucrose, pH 7.5) plus protease inhibitors and pelleted at 250*g* at 4 °C for 10 min. The supernatant was aspirated, and the cell pellet was resuspended in 600 μl of homogenization buffer. The cells were then mechanically homogenized by passing through a 25-gauge needle 20×. The homogenate was then pelleted at 1000*g* for 10 min at 4 °C to pellet the nuclear fraction. The supernatant was taken and further centrifuged at 45,000 rpm in a Beckman rotor (TLA 120.2) for 30 min at 4 °C to separate the cytosolic fraction from the endomembrane compartments.

The supernatant was discarded, and the pellet was then lysed in 200 μl of MNT buffer with protease inhibitors and sonicated for 20 s to fully resuspend the pellet. The lysis was then spun at 20,000*g* for 10 min at 4 °C to pellet the cellular debris. The sample was transferred to a new tube, where it was reduced with 40 mM DTT for 1 h and alkylated with iodoacetamide at 100 mM for 30 min while protected from light. The sample was then precipitated with five volumes of acetone overnight at −20 °C. The precipitate was centrifuged at 20,000*g* for 10 min at 4 °C to pellet the protein sample. The supernatant was aspirated and discarded, while the pellet was resuspended in 400 μl of 15 mM triethylamine bicarbonate (Thermo Fisher Scientific) and sonicated to fully suspend the pellet. The sample was incubated with 6 μg of Trypsin/Lys-C (Promega) overnight at 37 °C. After proteolysis, the sample was boiled at 95 °C to inactivate the proteases, and then the sample was treated with 2 μl of PNGaseF (NEB) overnight at 37 °C. After endoglycosidase treatment, peptides were quantified with the Pierce Quantitative Colorimetric Peptide Kit (Thermo Fisher Scientific).

10plex TMT (Thermo Fisher Scientific 0.8 mg) were resuspended in MS grade acetonitrile and was added to 40 μg of each digested peptide sample and incubated for 1 h at room temperature, per manufacturer’s instructions. Labeling was quenched by adding hydroxylamine to 0.25% and incubating for 15 min at room temperature. Labeled samples were pooled and fractionated utilizing the Pierce High pH Reversed-Phase Peptide Fractionation Kit (Thermo Fisher Scientific). Sample peptide concentration was then quantified using the same colorimetric assay as above.

### Quantitative LC-MS/MS acquisition

LC-MS analysis was performed, modified from (66) using an Easy-nLC 1000 nanoLC chromatography system interfaced to an Orbitrap Fusion mass spectrometer (Thermo Fisher Scientific). Samples were preconcentrated and desalted on to a C18 trap column prior to separation over a 180-min gradient from 0% to 50% mobile phase B (A: 0.1% formic acid in water, B: 0.1% formic acid in acetonitrile) at 300 nl/min flow rate with a 75 μm × 15 cm PepMap rapid separation liquid chromatography column (Thermo Fisher Scientific). MS parameters were as follows: ion spray voltage 2000V, survey scan MS1 120k resolution with a 2 s cycle time, interleaved with data-dependent ion trap MS/MS of highest intensity ions with charge state-dependent ion selection window (z = 2:1.2 *m/z*, z = 3:0.7 *m/z*, z = 4–6:0.5 *m/z*) and CID at 35% normalized collision energy. Additionally, the top five (z = 2) or 10 (z > 2) product ions were synchronously selected for higher collisional dissociation MS^3^ at normalized collision energy 65% with Orbitrap detection to generate TMT-tag intensities.

### Quantitative LC-MS/MS analysis

RAW data files were analyzed in Proteome Discoverer 2.5 (Thermo Fisher Scientific) using the SEQUEST search algorithm against *Homo sapiens* (SwissProt TaxID = 9606) database downloaded from uniprot.org. The search parameters used are as follows: 10 ppm precursor ion tolerance and 0.6 Da fragment ion tolerance; up to two missed cleavages were allowed; dynamic modification of methionine oxidation. Peptide matches were filtered to a protein false discovery rate of 5% using the Percolator algorithm. Peptides were assembled into proteins using maximum parsimony and only unique and razor peptides were retained for subsequent analysis. Protein quantitation based on TMT ion abundance was performed using a coisolation threshold of 75% and synchronous precursor selection match threshold 65%. Each TMT channel was normalized to total peptide amount and then abundance scaled to average 100% for all proteins. Protein ratios were calculated directly using grouped protein abundance and *p*-values calculated using unpaired *t* test.

## Data availability

All data is contained in the manuscript.

## Supporting information

This article contains supporting information (67, 68).

## Conflict of interest

The authors declare that they have no conflicts of interest with the contents of this article.

## Supporting information

Supporting Figures S1–S5 and references
