# Supplementary material for: TTC17 is an endoplasmic reticulum resident TPR-containing adaptor protein
Source: J Biol Chem. 2023 Nov 8;299(12):105450. doi: 10.1016/j.jbc.2023.105450 (PMC10783571; doi:10.1016/j.jbc.2023.105450)
Supplement: Supporting Figures S1–S5 and references [file mmc1.pdf]

SUPPORTIVE INFORMATION FOR

TTC17 is an endoplasmic reticulum resident TPR-containing adaptor protein

Nathan P. Canniff[1], Jill B. Graham[1], Kevin P. Guay[1], Daniel A. Lubicki[2], Stephen J. Eyles[1,2,3], Jennifer N. Rauch[1,2] and Daniel N. Hebert[1,2*]

From the [1]Program in Molecular and Cellular Biology; [2]Department of Biochemistry and Molecular Biology; [3]Institute for Applied Life Sciences, Mass Spectrometry Center, University of Massachusetts Amherst, USA

*To whom correspondence should be addressed: Daniel N. Hebert, Department of Biochemistry and Molecular Biology, University of Massachusetts, 240 Thatcher Road Amherst, MA 01003, Tel: (413) 545-0079, E-mail: dhebert@biochem.umass.edu

Supplementary methods

Radiolabeled pulse

Cells were plated and grown for 48 hr prior to pulse labelling. Cells were pulse labelled for 30 min or 60 min with EasyTag Express ^{35}S Protein Labelling Mix [^{35}S]-Cys/Met (PerkinElmer). After pulse labelling, the cells were washed twice with PBS on ice before cells were lysed with MNT lysis buffer. Samples were immunoprecipitated with IGF1R antibody and analyzed via SDS-PAGE followed by visualization by autoradiograph.

Quantitative RT-PCR

H4 WT and *TTC17*$^{-/-}$ cells were plated and grown for 48 hr. Cells were harvested and RNA was purified using the PureLink RNA Mini Kit (ThermoFisher). Purified mRNA was reverse transcribed using the Protoscript II Reverse Trasncriptase kit (NEB). qRT-PCRs were performed in 20 µL reactions using qPCR SyGreen Blue Mix Lo-ROX (PCRBiosystems) according to manufacturer's instructions. Quantification was determined using tubulin as the reference gene. Statistical analysis of three biological replicates were calculated between the WT and *TTC17*$^{-/-}$ cell lines and significance using and unpaired t-test.

Analysis of ER chaperones

HEK293 and H4 cells were cultured and grown on a 10-cm plate as described. Cells were lysed using MNT before the 30% of the lysate was precipitated with cold acetone and incubated overnight. The next day the samples were spun for 10 min at 20,000xg, 4 °C and the supernatants were aspirated. The pellets were dried overnight at 25 ˚C before being resuspending the next day with 80 μL of loading buffer (30 mM Tris-HCl, 9% SDS, 15% glycerol, and 0.05% bromophenol blue, pH 6.8) containing 100 mM dithiothreitol. Each tube was treated at 100 °C for 10 min and shaken vigorously for 10 min. 15 μL of each sample was resolved by 9% SDS-PAGE, transferred to a PVDF membrane and blotted using rabbit α-calnexin (Genetex, GTX109669), α-BiP (Cell Signaling, 3177S), α-calreticulin (LSBio, LS-C331242) or α-beta tubulin (Cell Signaling, 2128S) antibodies before labeling with an α-rabbit secondary antibody (LI-COR, 926-68071). Membranes were developed using a LI-COR Odessa and all statistical analyses were completed using GraphPad Prism v9.

Supplementary figures

A

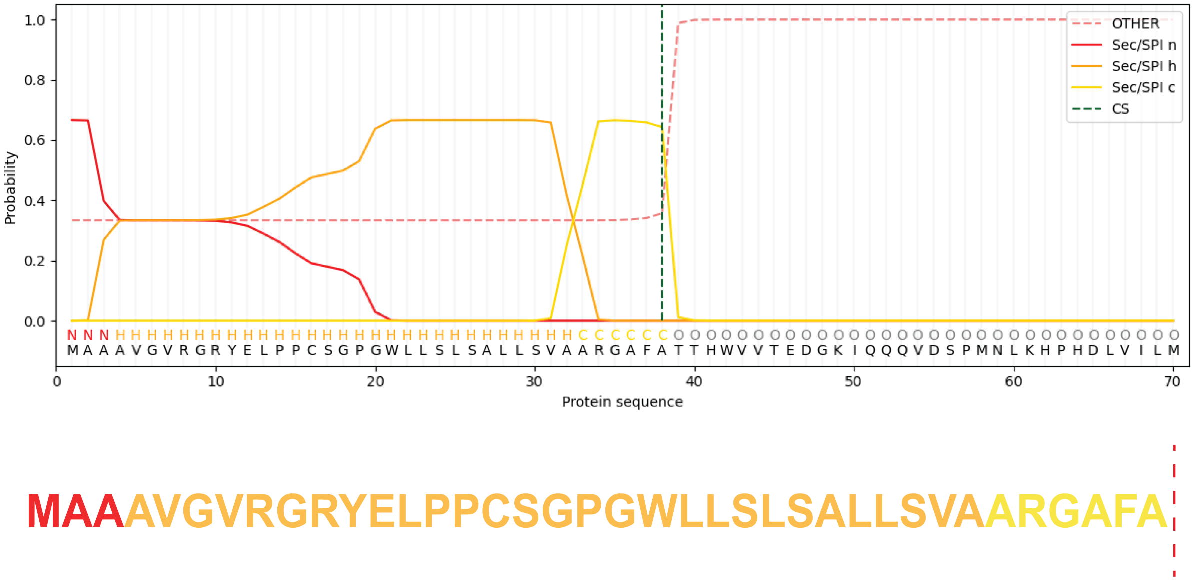

B

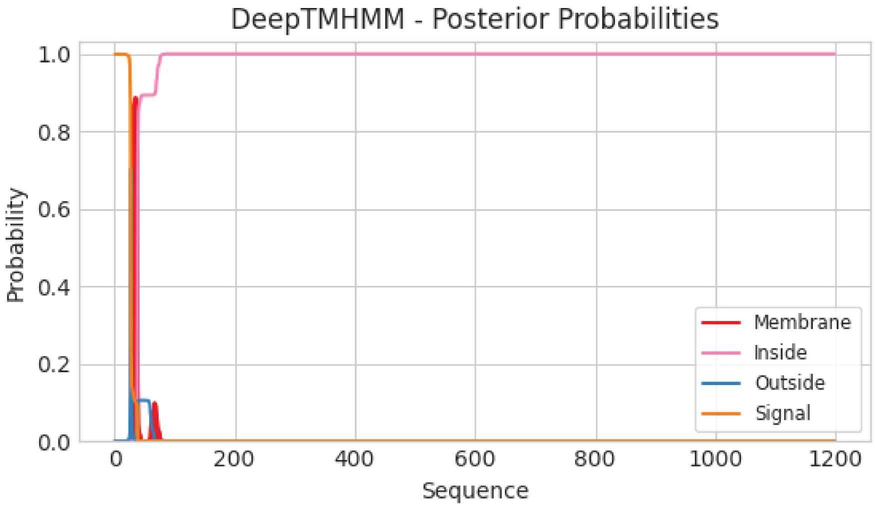

Figure S1. ***TTC17 is predicted to be a secretory protein via bioinformatic analysis*** **A**) TTC17 signal peptide prediction via SignalP6.0 with corresponding amino acid sequence below. **B**) TTC17 transmembrane domain prediction via DeepTMHMM algorithmic analysis.

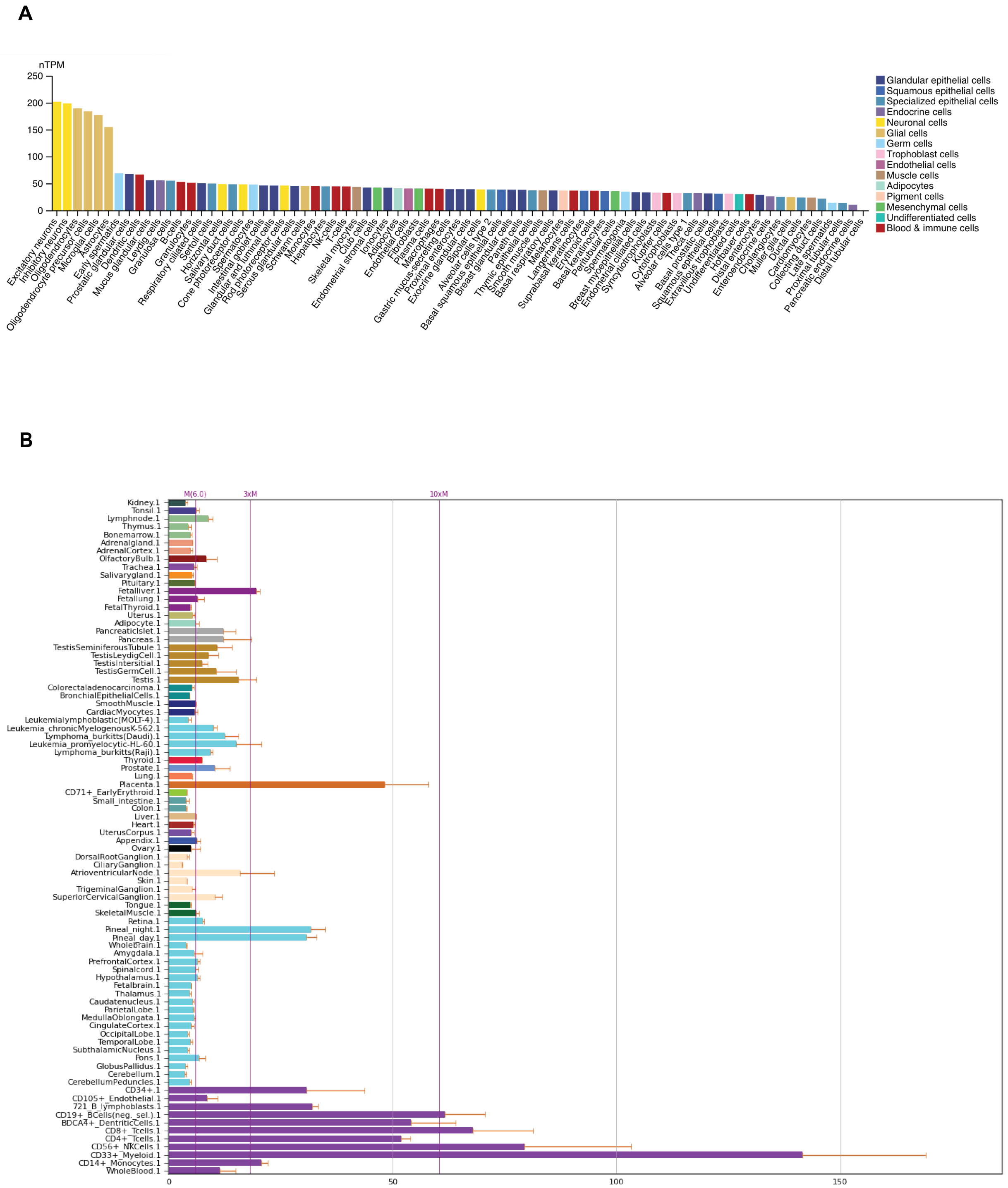

Figure S2. ***TTC17* *transcriptional expression varies widely between cell and tissue types*** **A**) *TTC17* transcript abundance across various tissues and cell types sourced from the Human Protein Atlas (Sjöstedt et al., 2020). *TTC17* abundance indicated to be highly enriched in neuronal and glial cell types. **B**) *TTC17* transcript abundance across various tissues and cell types sourced from the Human Protein Atlas (Wu et al., 2016). *TTC17* abundance indicated to be highly enriched in immune cells.

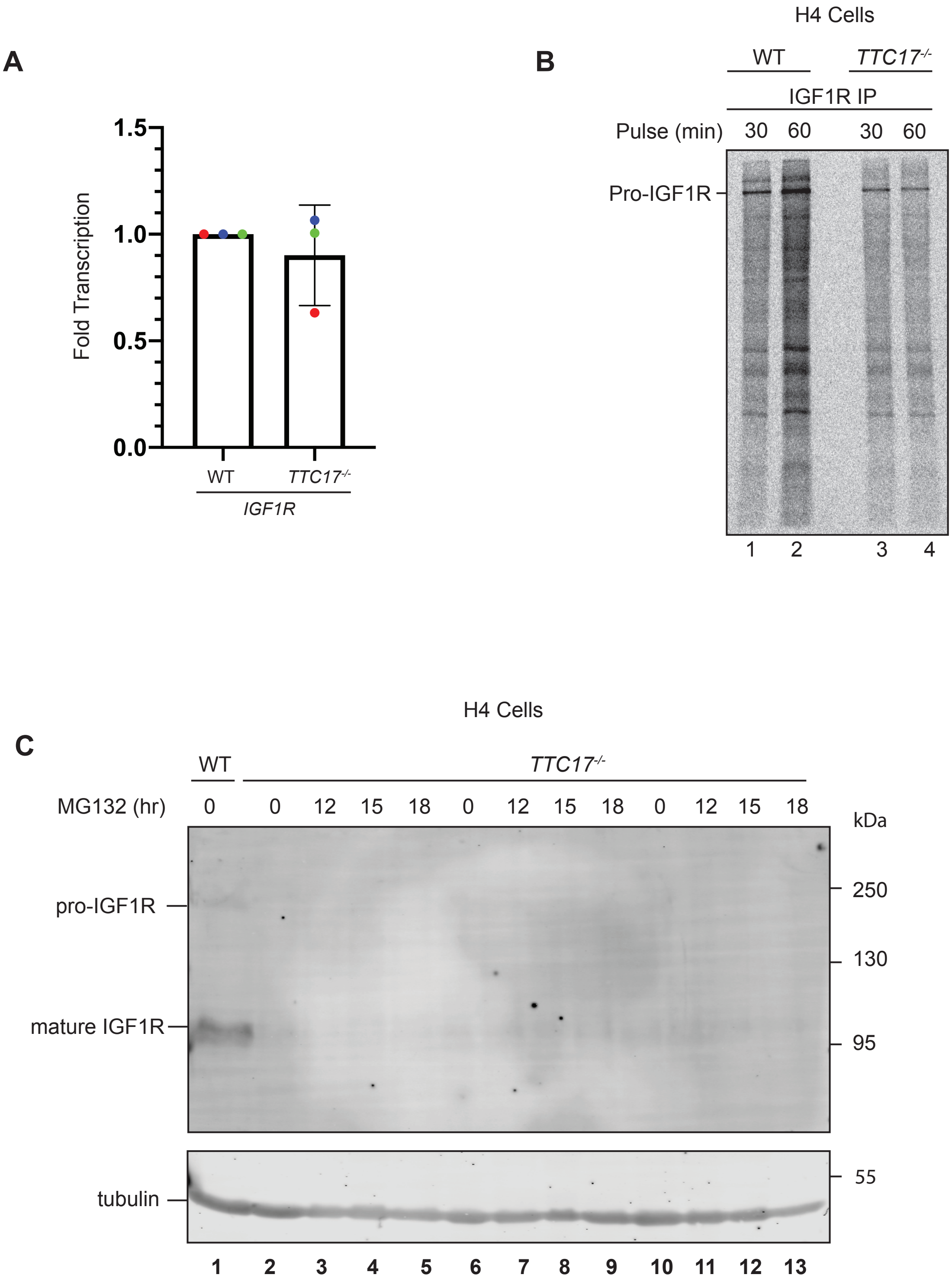
A
1.5
1.0
0.5
0.0
Fold Transcription
WT
TTC17-/-
IGF1R
B
H4 Cells
WT
TTC17-/-
IGF1R IP
Pulse (min) 30 60 30 60
Pro-IGF1R
1 2 3 4
C
H4 Cells
WT
TTC17-/-
MG132 (hr) 0 0 12 15 18 0 12 15 18 0 12 15 18
kDa
250
130
95
55
pro-IGF1R
mature IGF1R
tubulin
1 2 3 4 5 6 7 8 9 10 11 12 13

Figure S3. ***IGF1R transcription and translation are unperturbed in TTC17$^{-/-}$ cells.***
A) RNA was harvested from H4 WT and *TTC17*$^{-/-}$ cells. Purified mRNA was reverse transcribed to cDNA followed by qRT-PCR with appropriate primers, changes in gene expression were calculated using β-actin as a reference. Significance was determined across three independent experiments by unpaired t-test. **B**) Indicated cells were pulsed with [^{35}S]-Met/Cys for either 30 min or 1 hr. Cells were lysed and samples were immunoprecipitated using an anti-IGF1R antibody and resolved by reducing SDS-PAGE and imaged by autoradiograph. **C**) H4 WT or *TTC17*$^{-/-}$ cells were treated for the indicated times with MG132 before the cells were lysed. Samples were analyzed by SDS-PAGE followed by immunoblotting with an anti-IGF1R antibody. Beta-tubulin was used for a loading control.

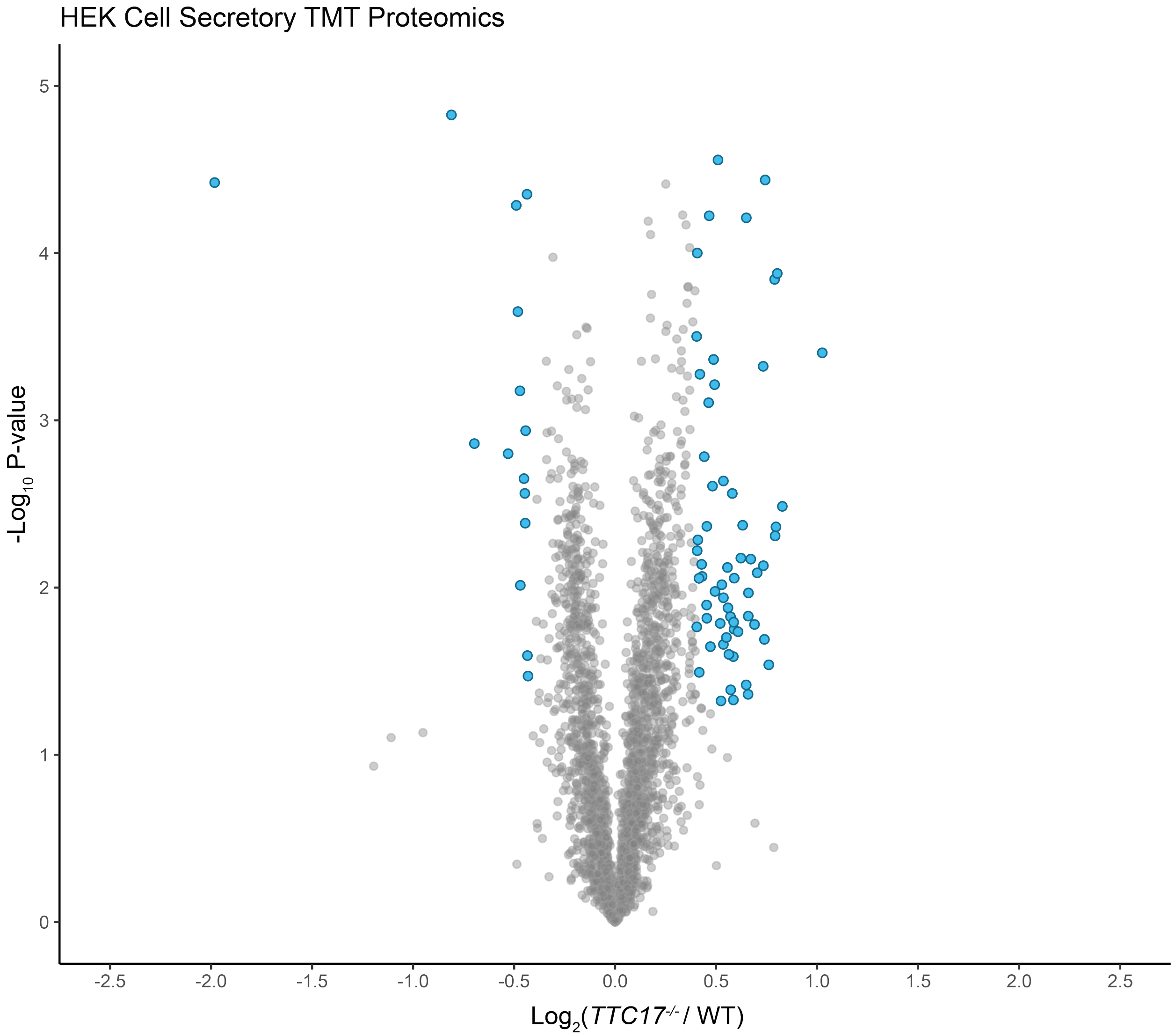

Figure S4. ***TTC17*$^{-/-}$ *in HEK cells affects a limited number of secretory proteins.*** Proteins were analyzed by dividing the quantification of the TMT label in the *TTC17*$^{-/-}$ cell line for each protein by that of the associated quantification of the TMT label in the WT cell line. Data is representative of three independent experiments. Highlighted (blue) points represent both P-value <0.05 and >0.4 or <-0.4 quantification enrichment.

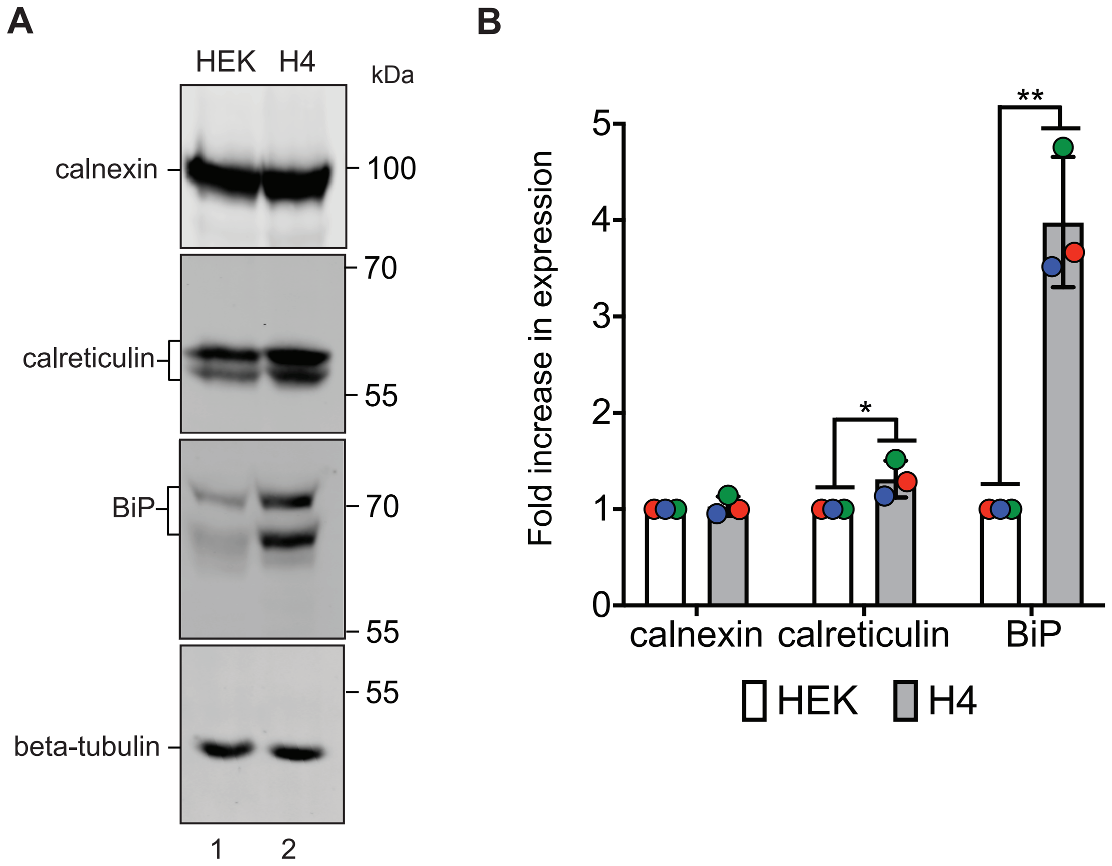

Figure S5. ***BiP is expressed significantly higher in H4 cells.*** **A)** HEK293 or H4 cells were lysed, and the resulting lysate was resolved by SDS-PAGE and analyzed by immunoblotting. Membranes were probed with an α-calnexin, α-calreticulin, and α-BiP to determine protein levels. Expression level was normalized to beta-tubulin. Immunoblots are representative of three independent biological replicates. **B**) Quantitation of protein levels from A. The signal intensity of protein from H4 cells were compared to HEK293, which was normalized to 1. Error bars represent standard deviation within the data set. * and ** indicate a P-value < 0.05 and 0.01, respectively.

Supplementary references

Sjöstedt, E., Zhong, W., Fagerberg, L., Karlsson, M., Mitsios, N., Adori, C., Oksvold, P., Edfors, F., Limiszewska, A., Hikmet, F., Huang, J., Du, Y., Lin, L., Dong, Z., Yang, L., Liu, X., Jiang, H., Xu, X., Wang, J., Yang, H., Bolund, L., Mardinoglu, A., Zhang, C., von Feilitzen, K., Lindskog, C., Pontén, F., Luo, Y., Hökfelt, T., Uhlén, M., Mulder, J., 2020. An atlas of the protein-coding genes in the human, pig, and mouse brain. Science 367, eaay5947. https://doi.org/10.1126/science.aay5947

Wu, C., Jin, X., Tsueng, G., Afrasiabi, C., Su, A.I., 2016. BioGPS: building your own mash-up of gene annotations and expression profiles. Nucleic Acids Research 44, D313–D316. https://doi.org/10.1093/nar/gkv1104
